# Myostatin Promotes Osteoclastogenesis by Regulating Ccdc50 Gene Expression and RANKL-Induced NF-κB and MAPK Pathways

**DOI:** 10.3389/fphar.2020.565163

**Published:** 2020-11-26

**Authors:** Xin Zhi, Qian Chen, Shaojun Song, Zhengrong Gu, Wenqiang Wei, Huiwen Chen, Xiao Chen, Weizong Weng, Qirong Zhou, Jin Cui, Liehu Cao

**Affiliations:** ^1^ Department of Orthopedics, PLA General Hospital, Beijing, China; ^2^ Basic Medical School, Naval Military Medical University, Shanghai, China; ^3^ Department of Emergency, General Hospital of Central Theather Command, Wuhan, China; ^4^ Department of Orthopedics, Shanghai Baoshan Luodian Hospital, Shanghai, China; ^5^ Department of Orthopedics Trauma, Shanghai Changhai Hospital, Naval Military Medical University, Shanghai, China

**Keywords:** osteoclastogenesis, myostatin, Ccdc50, Receptor activator of NF-κB ligand, NF-κB

## Abstract

Myostatin is a crucial cytokine that is widely present in skeletal muscle and that negatively regulates the growth and development of muscle cells. Recent research has shown that myostatin might play an essential role in bone metabolism. In RAW264.7 cells and bone marrow monocytes (BMMCs), myostatin activates the expression of the II type receptor ActR II B. Here, we report that myostatin significantly promoted RANKL/M-CSF-induced osteoclastogenesis and activated NF-κB and MAPK pathways *in vitro* via the Ccdc50 gene. Overexpression of myostatin promoted osteoclastogenesis and osteoclastogenesis-related markers including c-Src, MMP9, CTR, CK, and NFATc1. Specifically, myostatin increased the phosphorylation of Smad2, which led to the activation of NF-κB and MAPK pathways to activate osteoclastogenesis. Ccdc50 was identified as a gene whose expression was highly decreased in osteoclastogenesis upon myostatin treatment, and it could inhibit the function of myostatin in osteoclastogenesis by blocking NF-κB and MAPKs pathways. Our study indicates that myostatin is a promising candidate target for inhibiting RANKL-mediated osteoclastogenesis and might participate in therapy for osteoporosis, and that the Ccdc50 gene plays a significant role in the regulatory process.

## Introduction

Normal bone tissue is in a constant dynamic equilibrium state of bone formation and bone resorption. Bone formation is mediated by osteoblasts, while bone resorption is mainly mediated by osteoclasts (Kular et al., 2012). Differentiating from the monocyte-macrophage lineage, osteoclasts have essential physiological functions in bone resorption (Boyce et al., 2009). Receptor activator of NF-κB ligand (RANKL) stimulates osteoclast precursors and activates RANK by combining it with the co-effector M-CSF (Mizukami et al., 2002), which can promote bone resorption in the process of normal or pathological bone remodeling.

Nuclear factor-kappa B (NF-κB) belongs to a family including p65, p52, p50, RelB, and c-Rel, and positively regulates the expression of many genes related to inflammation and other reactions (Courtois and Gilmore, 2006). Osteoclast precursor cells rapidly initiate the classical NF-κB pathway in response to RANKL (Asagiri et al., 2005). After the activation of the classical NF-κB pathway, the non-classical NF-κB pathway is activated and lasts for several hours (Boyce et al., 2015). The NF-κB pathway is activated as TRAF I is recruited, and then the nuclear factor of activated T-cells cytoplasmic 2 (NFATc2) binds to NF-κB to induce activation and expansion of nuclear factor of activated T-cells cytoplasmic 1 (NFATc1) (Takayanagi et al., 2002). NFATc1 promotes the expression of genes related to the proliferation and differentiation of osteoclasts. The induction of NFATc1/NFAT2 and the classical and non-classical NF-κB pathways are necessary for transcription and translation of osteoclastogenesis-critical genes under the induction of RANKL/RANK-mediated activation. In this way, monocyte-derived macrophages fuse and differentiate into osteoclasts with mature osteolytic function (Walsh and Choi, 2014). The mitogen-activated protein kinase (MAPK) pathway represents a series of serine-threonine kinases in eukaryotes, and functions by phosphorylating other cytoplasmic proteins and directly regulating transcription after translocating from the cytoplasm to the nucleus (Hagemann and Blank, 2001). The MAPK pathway is organized into three-tiered cascades comprising three types of molecules: MAPKs, MAPK kinases (MAPKK or MEK), and MAPKK kinases (MAPKKK or MEKK) (Lee et al., 2018). Generic MAPK signaling includes the c-Jun N-terminal kinase (JNK), extracellular signal-related kinase (ERK), and p38 (Sun et al., 2015). JNK and p38 mediate osteoclast precursor proliferation and osteoclast differentiation when induced by RANKL and M-CSF, while ERK accelerates the proliferation of osteoclast precursors via the M-CSF/c-Fms axis (Lee et al., 2018). Moreover, p38 phosphorylates its transactivation domain with the co-stimulation of c-fos both *in vivo* and *in vitro* (Tanos et al., 2005).

Myostatin, which is also called GDF-8, is a member of the TGF-β family. Previous research has shown that myostatin is synthesized by muscle cells and is a critical autocrine/paracrine inhibitor of skeletal muscle growth (Nissinen et al., 2016). The binding of myostatin and the II type receptor ActR II B occurs on the cytomembrane, then Smad2/3-mediated transcription is activated and inhibits muscle protein synthesis (Han et al., 2013). A recent study showed that bone formation is enhanced when the expression of myostatin is deficient (Hamrick, 2003; Bialek et al., 2014). RANKL-mediated osteoclastogenesis is accelerated by myostatin under transcription factor SMAD2-dependent regulation of NFATc1 (Dankbar et al., 2015). In the present study, we investigated the mechanism by which myostatin regulates the RANKL-induced downstream pathway.

## Method

### Reagents and Inhibitor

Myostatin was provided by Phoenix pep (San Francisco, CA, United States). RANKL was provided by Raybiotech (Guangzhou, China). The Smad2 inhibitor LY209761 was supplied by Selleck (Houston, TX, United States).

### Osteoclastogenesis Assay *In Vitro*

RAW264.7 cells were provided by the Chinese Academy of Sciences (Shanghai, China). BMMCs were separated by flushing the bone marrow of femurs from 4-week-old C57BL/6 mice. Cells were seeded into 24-well plates at a density of 8 × 10^3^ cells/well. Cells of the third generation were induced with RANKL (50 ng/ml) and M-CSF (30 ng/ml) with or without myostatin treatment (30 ng/ml). DMEM medium with low-glucose used to culture RAW264.7 cells and BMMCs was mixed with 10% fetal bovine serum and 1% penicillin-streptomycin as supplied by the Experiment Center of Changhai Hospital (Shanghai, China). Cells were cultured at 37°C and 5% CO_2_.

### TRAP Staining

Osteoclasts were stained by a TRAP staining kit from Sigma-Aldrich (St. Louis, MO, United States), and the procedure was strictly according to the protocol of the manufacturer. The number and size of osteoclasts were observed under an upright metallurgical microscope. Huge fused multinucleated (greater than or equal to three) cells were considered as osteoclasts.

### Immunofluorescence Staining

The localization of ActR II B in RAW264.7 cells and BMMCs was observed by immunofluorescence. Cell medium was removed and replaced with 4% paraformaldehyde to fix cell morphology. Then paraformaldehyde was washed out by Triton X-100, and the fixed cells were incubated with myostatin conjugated with fluorescein. The cellular localization of ActR II B was observed by using protocols as previously described (Wen et al., 2019).

### Western Blotting

Cells were collected and lyzed for extracting protein by the M-PER mammalian protein extraction reagent supplied by Pierce (Rockford, IL, United States). The concentration of protein was evaluated by a bicinchoninic acid (BCA) protein assay kit supplied by Pierce. Equal amounts of protein were added into SDS-PAGE gels with 10 μg protein per lane. The blots were probed with a monoclonal antibody against human anti-ActR II B (1:100), anti-c-Src (1:200), anti-MMP-9 (1:200), anti-CTR (1:200), anti-CK (1:200), anti-P-Smad2 (1:200), anti-Smad2 (1:200), anti-P-p65, anti-p65 (1:300) (1:500), anti-P-p50 (1:400), anti-p50 (1:250), anti-P-ERK (1:400), anti-ERK (1:200), anti-P-JNK (1:400), anti-JNK (1:500), anti-P-p38 (1:300), anti-p38 (1:300), anti-P-c-fos (1:400), anti-c-fos (1:400), and anti-β-actin (1:1000) from Santa Cruz (TX, United States), and the secondary HRP-conjugated anti-mouse/rabbit antibody was purchased from Santa Cruz. After washing with TBST, chemiluminescence was performed to detect the bands. β-actin was added as an internal control for eliminating errors in protein quantification and loading. Proteins were transferred onto nitrocellulose membranes after electrophoresis. After transfer, primary antibodies were used for incubation, and then secondary antibodies were used for exposure and development. Finally, film exposure was performed, and images were analyzed by Image-Pro Plus software. All the experiments were detected three times, and the average was calculated.

### RT-PCR

Total RNA was extracted from RAW264.7 cells to measure the expressions of ActR II B, c-Src, MMP-9, CTR, NFATc1, and Ccdc50, using Trizol reagent from Invitrogen (Carlsbad, CA, United States) according to the manufacturer’s protocol. Reverse transcription was performed on extracted RNA to synthesize cDNAs by using a SYBR1 Premix Dimmer Eraser kit (TaKaRa Biotechnology, Otsu, Japan). Realtime PCR was then executed using SYBR^®^ Premix Ex TaqTM II (Tli RNaseH Plus) purchased from TaKaRa Biotechnology (Dalian, China) and detected by an ABI 7500 Sequencing Detection System from Applied Biosystems (Foster City, CA, United States). The primer sequences used were as follows: MMP-9: (F) GGA​CCC​GAA​GCG​GAC​ATT​G; (R) CGT​CGT​CGA​AAT​GGG​CAT​CT, C-Src: (F) CAA​CTT​CGG​CAC​AGC​AAC; (R) TCA​GAC​ACC​AGC​ACA​TTC​C, ActRIIB: (F) GCT​CCC​TCA​CGG​ATT​ACC; (R) CAC​GAC​ACC​ACG​GCA​CAT, CTR: (F) CCT​GGT​TGA​GGT​TGT​GCC; (R) GCG​TTG​CTC​GTC​GGT​AAA, NFATc1: (F) ACC​ACT​CCA​CCC​ACT​TCT​G; (R) GCT​GCC​TTC​CGT​CTC​ATA​G, Ccdc50: (R) AAA​GAG​GGT​GAT​GAA​GCA; (F) ATG​GAA​GCC​TTT​CTG​TGA.

### Pit-formation Assays

RAW264.7 cells and BMMCs for detecting differentiation were seeded on hydroxyapatite-coated surfaces of biomimetic synthetic bone from Corning (Bedford, MA, United States). Culture medium was changed on day 3. After incubation for 7 days, the medium was washed out with PBS and dried in the air at room temperature for 5 h. A representative region of osteoclast-resorbing pits was photographed using a light microscope (BX53) provided by Olympus (Tokyo, Japan). Further research and analysis were carried out by Image-Pro Plus software.

### CHIP Assay

RAW264.7 cells treated with myostatin were subjected to chromatin immunoprecipitation assays by a CHIP kit (EpiGentek, NY, United States). In brief, cells cultured in 24-well plates were fixed by formaldehyde. Lysates digested by cell lysis buffer were collected, followed by mixing with Smad2-specific IgG. Then the mixture was incubated at room temperature overnight for 12 h. Protein Agarose was added for combining antibody-target protein-DNA complexes. The supernatant was discarded after centrifugation at 4°C for 30 min at 3,000 *g*, followed by washing to obtain the target protein-DNA complex. Then the crosslinked purified DNA was removed. Further analysis was conducted by qRT-PCR. Nonprobe-based analyses were performed using SYBR^®^ green as an intercalating dye for target detection. Based on the quantification of housekeeping genes, the procedures and conditions of the genes of interest had internal controls to prevent influence by the experimental conditions. Enrichment analysis for differentially expressed genes that were up- or down-regulated was performed by Gene Ontology (GO) functional and Kyoto Encyclopedia of Genes and Genomes (KEGG) analysis.

### Lentivirus Infection of RAW264.7 Cells

An siRNA sequence with a target sequence homologous to Ccdc50 was chemically synthesized to inhibit the transcription of mouse Ccdc50. The artificially synthesized oligonucleotide templates were cloned into the linear pSIH-H1-copGFP siRNA vector (System Biosciences, Palo Alto, CA, United States) to form pSIH-shRNA-Ccdc50. A negative control (NC) was originated from an invalid siRNA sequence, and the negative control expression vector pSIH-NC was constructed. DNA of Ccdc50 was obtained from total mouse RNA extracted from different tissues with treatment by Trizol (Invitrogen) followed by reverse transcription and PCR. Then, the Ccdc50 expression vector pcDH-Ccdc50 was constructed after the artificial DNA was cloned into pcDH-GFP Lentivector (CD511A-1, System Biosciences).

The plasmids (pSIH-NC, pSIH-shRNA-Ccdc50, and pcDH-Ccdc50) were transfected into 293T cells together with the packaging vector (System Biosciences) to produce lentiviruses Lv-NC, Lv-shRNA-Ccdc50, and Lv-Ccdc50. The harvested virus suspensions were concentrated by ultrafiltration and purification (Benskey and Manfredsson, 2016). RAW264.7 cell suspensions digested by trypsinization were divided into four groups and seeded on six-well plates, then cultured at 37°C and 5% CO_2_. The groups were 1) cells infected with Lv-NC; 2) cells infected with Lv-shRNA Ccdc50; 3) cells infected with Lv-Ccdc50; and 4) control group (not infected). After culturing for 12 h, the pre-configured lentiviral solution at MOI = 20 was added into the corresponding wells and mixed with medium. After 3 days, the cells were digested and extracted for total protein, and transfection efficiency was determined after the measurement of the amount of Ccdc50 by western blotting (Sakuma et al., 2012).

### Statistical Analysis

All results were tested by a homogeneity of variance test and two samples independent *t* test by SPSS Statistics supplied by IBM (New York, United States). *p* < 0.05 was considered to be statistically significant.

## Results

### The Expression of ActR II B Increases During Osteoclastogenesis

**FIGURE 1:**
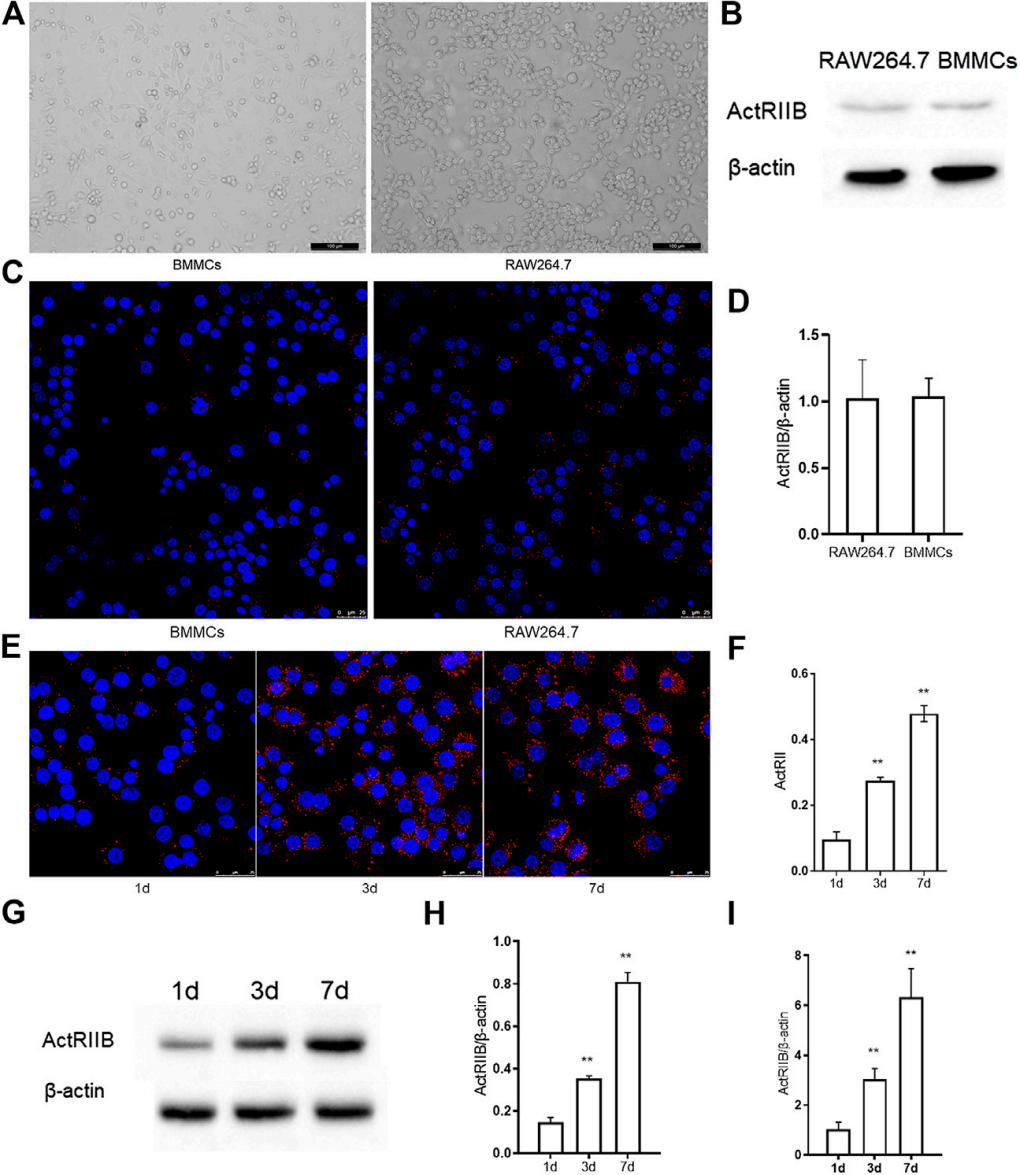
The expression of ActR II B increases during osteoclastogenesis. **(A)** Cells were visualized by an inverted optical microscope. **(B)** ActR II B protein was extracted for analysis by western blot. **(C)** Immunofluorescence staining of ActR II B in RAW264.7 cells and BMMCs. **(D)** RT-PCR was used to determine the expression of ActR II B. **(E,F)** Immunofluorescence staining of ActR II B in RAW264.7 cells and BMMCs on days 1, 3, and 7. **(G,H)** Lyzed protein from induced RAW264.7 cells was analyzed by western blotting. **(I)** Total RNA was analyzed by RT-PCR to examine the expression of mRNA. **p < 0.01.

To research the function of myostatin in osteoclastogenesis *in vitro*, we first detected the expression of ActR II B, which is a receptor of myostatin. We chose RAW264.7 and BMMCs, which can be induced to form osteoclasts (Battaglino et al., 2002; Takayanagi, 2007) *in vitro* (Figure 1A). Western blotting indicated that ActR II B had low expression in RAW264.7 cells and BMMCs before the induction of osteoclast differentiation (Figure 1B). Immunofluorescence staining and RT-PCR also indicated that little ActR II B was expressed in RAW264.7 cells and BMMCs (Figures 1C,D). Then, BMMCs were treated with RANKL and M-CSF, while RAW264.7 cells were treated with RANKL for induction (Bharti et al., 2004; Lee et al., 2006). ActR II B significantly increased after induction by RANKL (Figures 1E,F). Western blotting and RT-PCR showed similar results (Figures 1G–I).

### Myostatin Promotes Osteoclastogenesis and Osteoclast Function

**FIGURE 2:**
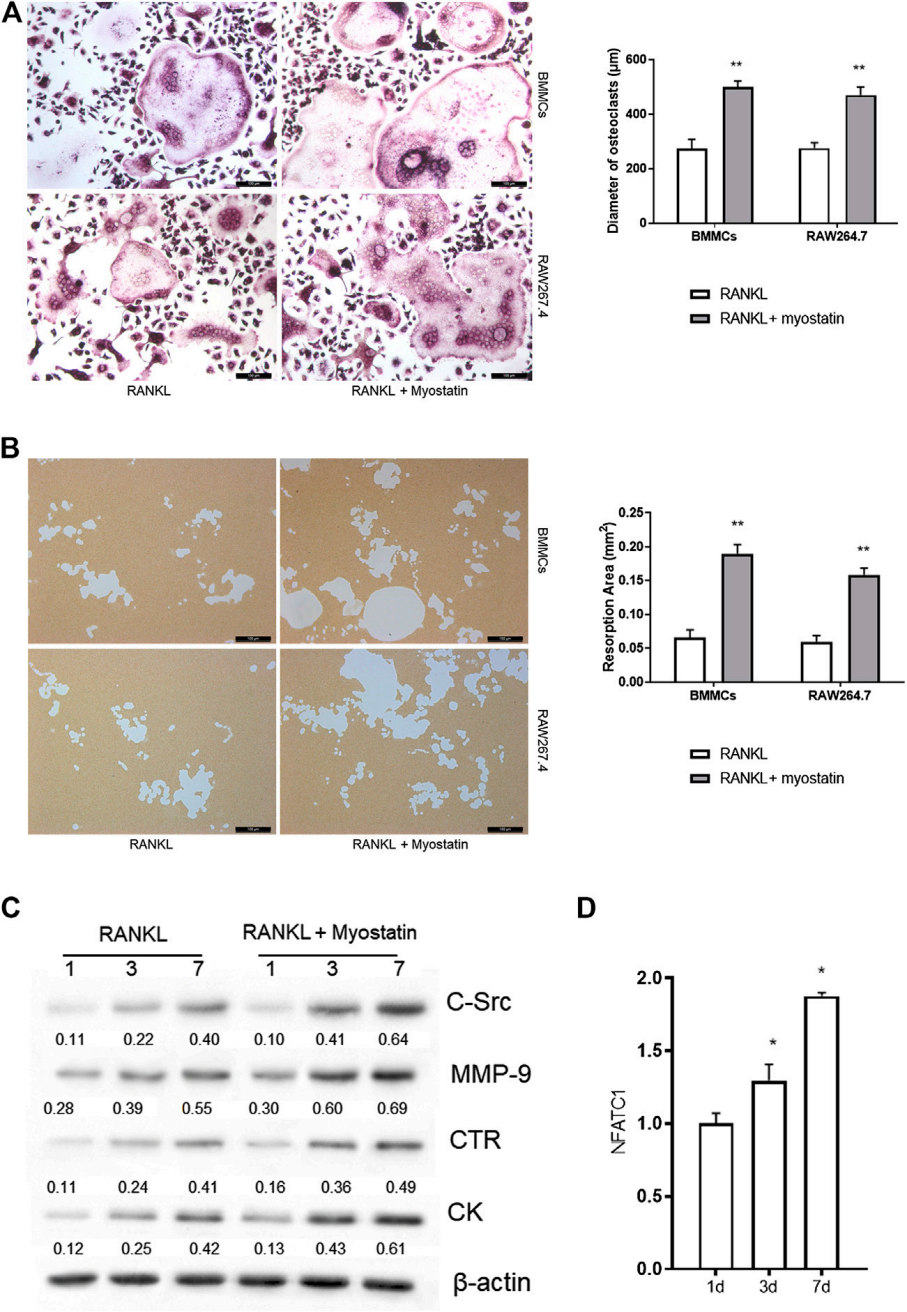
Myostatin promotes osteoclastogenesis and osteoclast function. **(A)** TRAP staining of osteoclasts (Scalebar, 100 μm). **(B)** Representative images of resorption pits localized on biomimetic synthetic bone; the resorptive area was analyzed in the histogram (Scale bar, 100 μm). **(C)** Expressions of C-Src, MMP-9, CTR, cathepsin K, and β-actin as analyzed by western blotting. **(D)** The total RNA of NFATc1 was analyzed by RT-PCR. **p < 0.01.

During the differentiation of osteoclasts, the expression of ActR II B was upregulated. We next examined whether myostatin promoted osteoclast precursors to differentiate into mature osteoclasts. We found that the size of osteoclasts was larger in the presence of myostatin than in its absence in the process of RANKL-induced osteoclastogenesis (Figure 2A). To further verify the mechanism by which myostatin influences the function of osteoclasts, we performed bone resorption experiments. When BMMCs and RAW264.7 were induced by RANKL, the surface of the biomimetic synthetic bone was resorbed, and the resorbed area was further expanded with the addition of myostatin (Figure 2B). Meanwhile, treatment with myostatin significantly increased the expression of osteoclast-related markers, including C-Src, MMP-9, CTR, and cathepsin K (Figure 2C; Supplementary Figure S1). We next assessed the effect of myostatin on the expression of NFATc1. The results showed an increase of NFATc1 transcription via RANKL induction, and this was further promoted by myostatin treatment (Figure 2D).

### Myostatin Promotes the Differentiation of Osteoclasts by Activating Smad2 and Activates the NF-κB and MAPK Pathways

**FIGURE 3:**
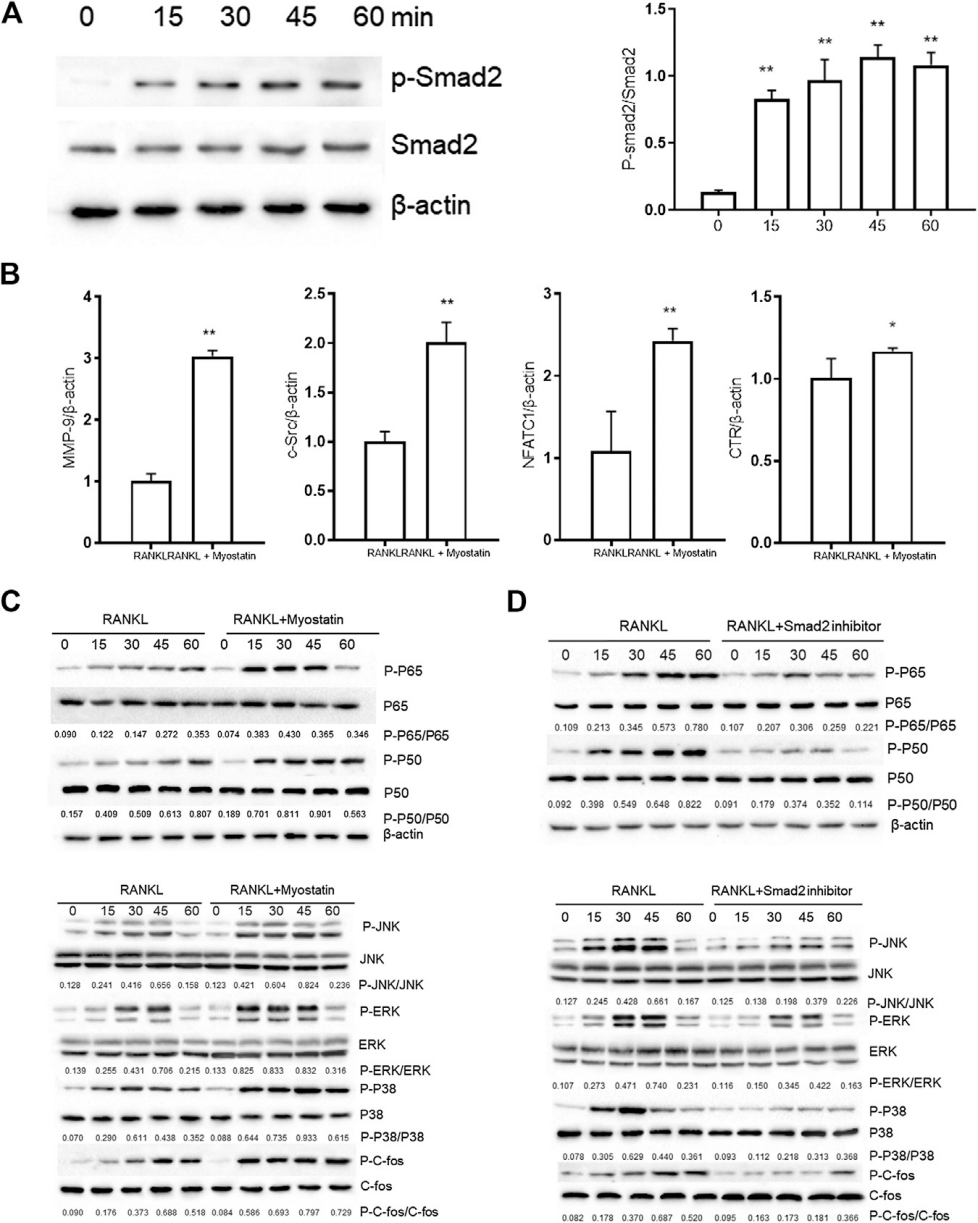
Myostatin promotes the differentiation of osteoclasts by activating Smad2 and activates NF-κB and MAPK pathways. **(A)** P-Smad2 and Smad2 were detected by western blot. **(B)** Total RNA from RAW264.7 cells treated with or without myostatin for 24 h was analyzed by RT-PCR to detect the expressions of CTR, C-SRC, MMP-9, and NFATc1. **(C)** The expressions of P-p65/p65, P-p50/p50, P-JNK/JNK, P-ERK/ERK, P-p38/p38, and P-c-fos/c-fos were analyzed by western blotting. **(D)** RAW264.7 cells were cultured with RANKL and M-CSF in the presence or absence of LY209761 and collected at 0, 15, 30, 45, and 60 min. The expressions of P-p65/p65, P-p50/p50, P-JNK/JNK, P-ERK/ERK, P-p38/p38, and P-c-fos/c-fos were analyzed by western blot. *p < 0.05, **p < 0.01.

The phosphorylation of Smad2 increased in RAW264.7 cells with myostatin treatment (Figure 3A). In addition, treatment with myostatin significantly elevated the expressions of CTR, C-Src, MMP-9, and NFATc1, which are related to osteoclastogenesis (Figure 3B). NF-κB and MAPK pathways are crucial for osteoclast differentiation. The phosphorylation of p65 and p50, which is related to the activation of NF-κB, was enhanced by myostatin within 60 min during the differentiation process of RAW264.7 cells. In addition, the phosphorylation of JNK, ERK, p38, and c-fos were enhanced by myostatin, indicating that the MAPK pathway was activated by myostatin (Figure 3C; Supplementary Figure S2). However, the phosphorylation of p65, p50, JNK, ERK, p38, and c-fos were suppressed with the addition of LY209761, which is an inhibitor of Smad2 (Figure 3D; Supplementary Figure S3), indicating that NF-κB and MAPK pathways were regulated by Smad2.

### Functional Enrichment Analysis of Differentially Expressed Genes

**FIGURE 4:**
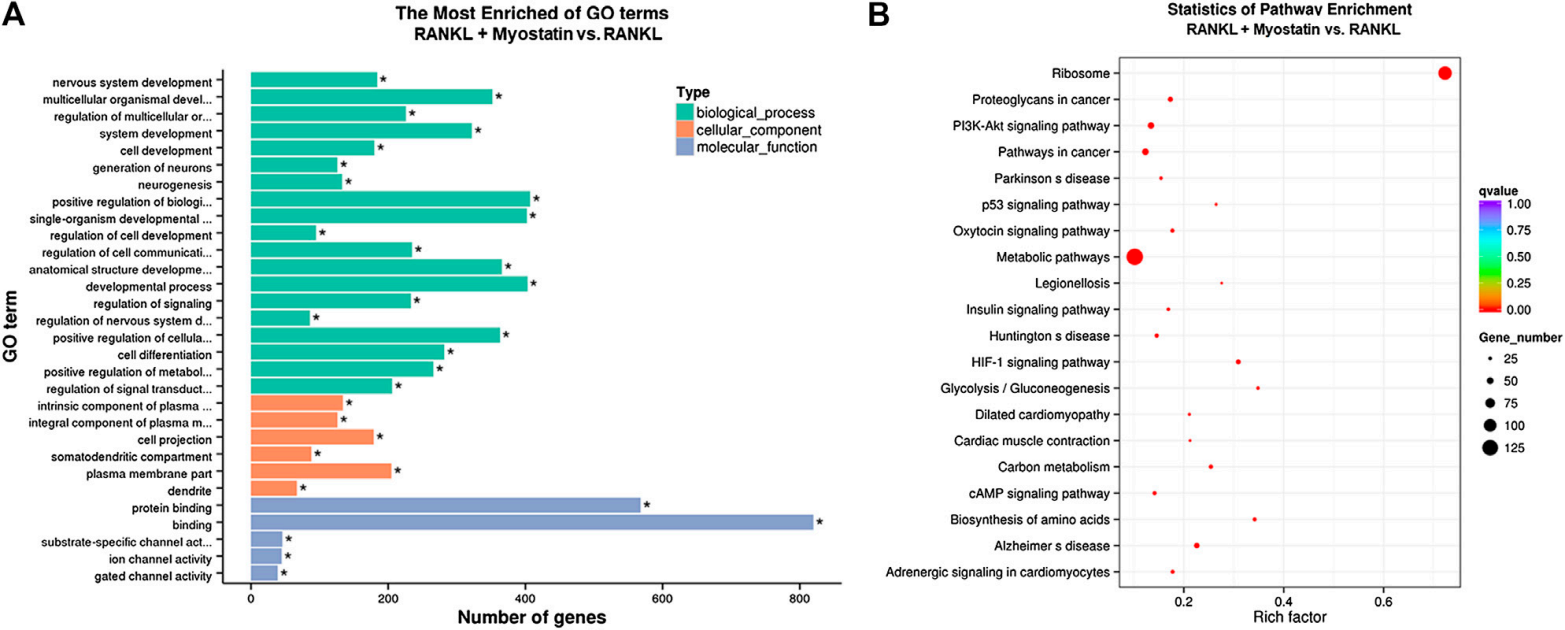
Functional enrichment analysis of differentially expressed genes. **(A)** Differentially expressed genes in osteoclastogenesis were researched by GO analysis. **(B)** Differentially expressed genes in osteoclastogenesis were researched by KEGG analysis.

To illuminate the biological functions and signaling pathways of osteoclastogenesis after treatment with myostatin, GO functional enrichment and KEGG enrichment analyses were carried out. The differentially expressed genes (DEGs) were mainly involved in 30 GO terms (Figure 4A) and 20 pathways (Figure 4B). GO enrichment was markedly associated with biological processes, cellular components, and molecular functions. Notably, the number of pathways involved in biological processes was the most, including positive regulation of biological processes, single-organism developmental processes, and developing process. KEGG pathway enrichment revealed remarkable involvement of myostatin in pathways including metabolic pathways, ribosomes, pathways in cancer, and the PI3K-Akt signaling pathway. To investigate specific genes whose expression changed greatly in osteoclastogenesis under the effect of myostatin, we listed 10 relatively high expression genes (Table 1) and relatively 10 low expression genes (Table 2) after treatment with myostatin in osteoclastogenesis.

**TABLE 1:** The high expression gene in osteoclastogenesis after treated with myostatin.

	Overlap gene	Overlap symbol	Fold change
1	ENSMUSG00000031142	Cacna1f	3.32089
2	ENSMUSG00000039068	Zzz3	3.04928
3	ENSMUSG00000056476	Med12l	3.03803
4	ENSMUSG00000021509	Il9	3.00579
5	ENSMUSG00000027261	Hao1	2.96474
6	ENSMUSG00000096915	Btbd35f6	2.52734
7	ENSMUSG00000026885	Ttll11	2.40835
8	ENSMUSG00000075604	Cyp11b1	2.32850
9	ENSMUSG00000041710	Trpc5	2.25990
10	ENSMUSG00000022263	Trio	2.19536

**TABLE 2:** The low expression gene in osteoclastogenesis after treated with myostatin.

	Overlap gene	Overlap symbol	Fold change
1	ENSMUSG00000054321	Taf4b	−4.15787
2	ENSMUSG00000026656	Fcgr2b	−3.96007
3	ENSMUSG00000022350	Sqle	−3.91942
4	ENSMUSG00000043314	Rik	−3.86284
5	ENSMUSG00000059366	Olfr	−3.82993
6	ENSMUSG00000024979	Tectb	−3.82828
7	ENSMUSG00000046364	Rpl27a	−3.81251
8	ENSMUSG00000030492	Slc7a9	−3.5715
9	ENSMUSG00000038127	Ccdc50	−3.53979
10	ENSMUSG00000039745	Htatip2	−3.45192

### Ccdc50 Regulates Osteoclastogenesis

**FIGURE 5:**
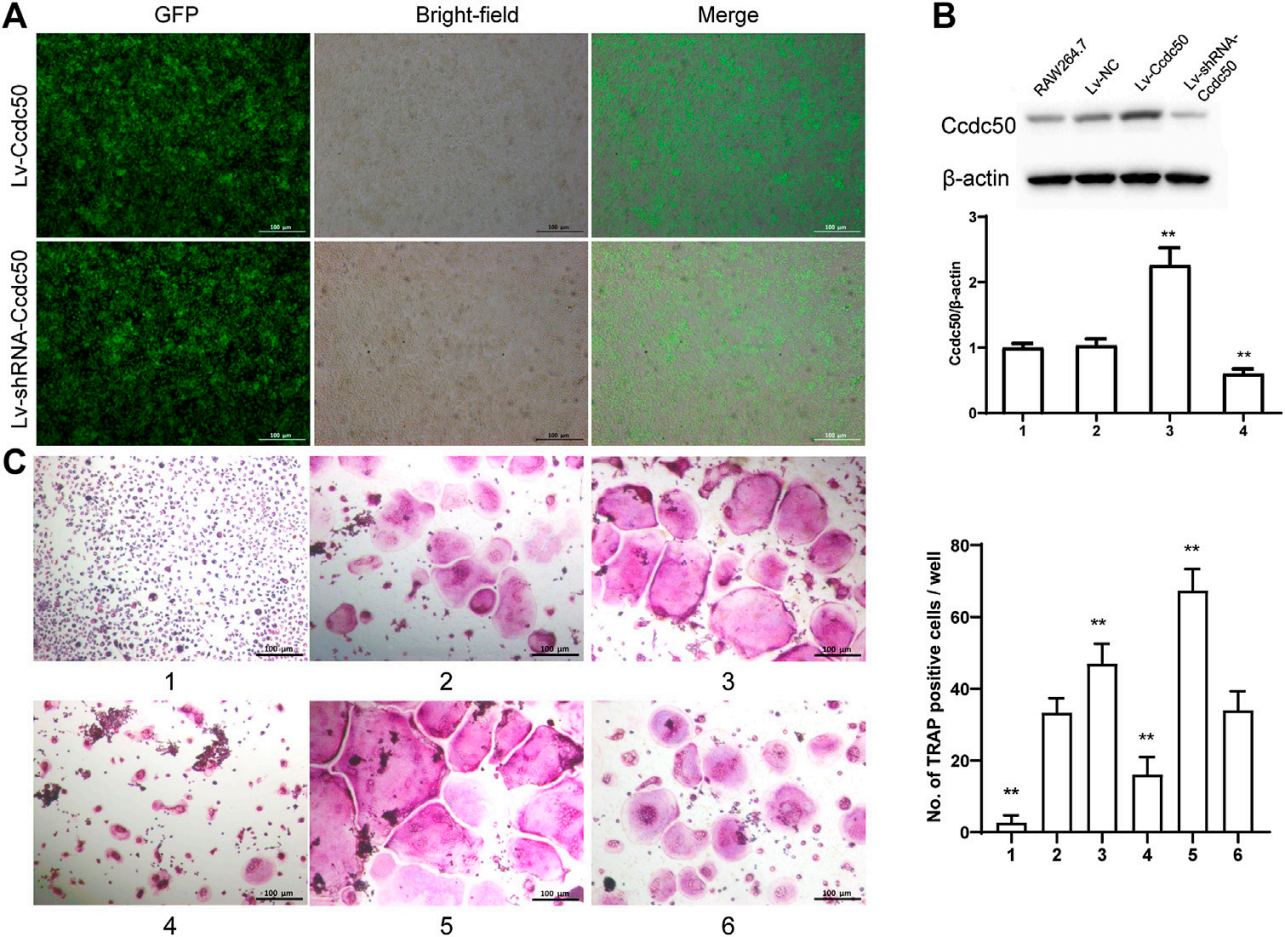
Myostatin regulates osteoclastogenesis under the regulation of Ccdc50. **(A)** The localization of Ccdc50 expressed by lentivirus after 72 h was ascertained by fluorescence staining. **(B)** The expression of Ccdc50 of four groups were detected by western blot. **(C)** Representative images and quantitative analysis of RAW264.7 cells cultured in the six groups and stained for TRAP. 1) RAW264.7 cells. 2) RAW264.7 cells infected with Lv-NC, induced with M-CSF, RANKL. 3) RAW264.7 cells infected with Lv-NC, induced with M-CSF, RANKL, and 30 ng/ml myostatin. 4) RAW264.7 cells infected with Lv-Ccdc50, induced with M-CSF, RANKL. 5) RAW264.7 cells infected with Lv-shRNA-Ccdc50, induced with M-CSF, RANKL, and 30 ng/ml myostatin; 6) RAW264.7 cells infected with Lv-Ccdc50, induced with M-CSF, RANKL, and 30 ng/ml myostatin.

We selected the Ccdc50 gene as a target gene that regulates osteoclastogenesis under the effect of myostatin. We first investigated whether osteoclastogenesis was regulated by Ccdc50. Ccdc50 levels were measured in RAW264.7 cells infected with Lv-Ccdc50, Lv-shRNA-Ccdc50, and Lv-NC. Ccdc50 was successfully overexpressed and silenced (Figures 5A,B). To further investigate whether myostatin could enhance osteoclastogenesis together with Ccdc50, RAW264.7 cells were treated with Lv-NC, Lv-shRNA-Ccdc50, or Lv-Ccdc50 in the presence of RANKL, then treated with 30 ng/ml myostatin. TRAP staining indicated that overexpressed Ccdc50 suppressed osteoclastogenesis in the presence of myostatin (Figure 5C). The effects of osteoclastogenesis were weakened when Ccdc50 was overexpressed, while when Ccdc50 was not expressed, the process of osteoclastogenesis was enhanced.

### Ccdc50 Regulates PI3K/Akt, MAPK, and NF-κB Pathways

**FIGURE 6:**
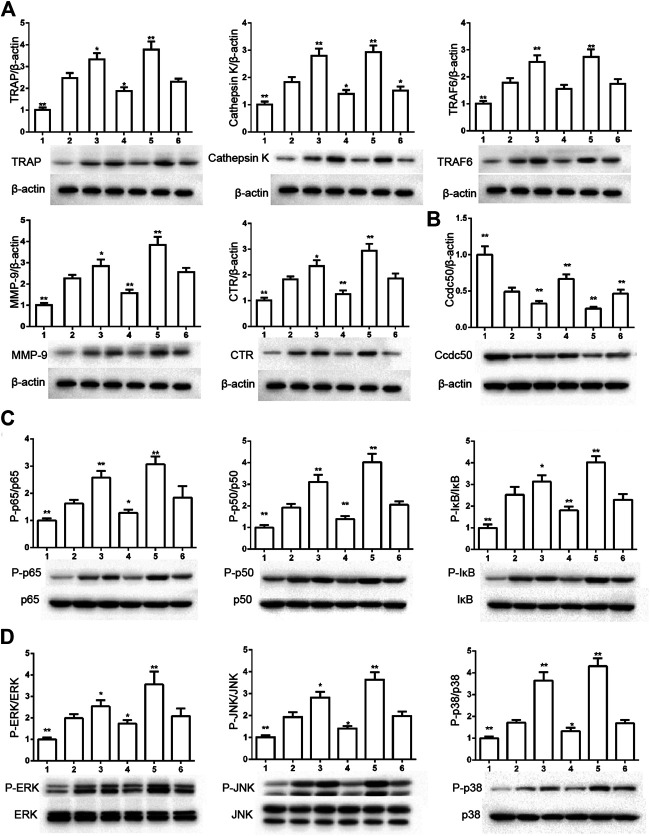
Ccdc50 regulates NF-κB, and MAPK pathways. **(A)** The expressions of TRAP, cathepsin K, TRAF6, MMP9, and CTR were analyzed by western blot. **(B)** Changes in Ccdc50 in osteoclastogenesis. **(C)** Phosphorylation of p65, p50, and IκB as analyzed by western blot. **(D)** Phosphorylation of JNK, ERK, and p38 as analyzed by western blot. 1) RAW264.7 cells. 2) RAW264.7 cells infected with Lv-NC, induced with M-CSF, RANKL. 3) RAW264.7 cells infected with Lv-NC, induced with M-CSF, RANKL, and 30 ng/ml myostatin. 4) RAW264.7 cells infected with Lv-Ccdc50, induced with M-CSF, RANKL. 5) RAW264.7 cells infected with Lv-shRNA-Ccdc50, induced with M-CSF, RANKL, and 30 ng/ml myostatin; 6) RAW264.7 cells infected with Lv-Ccdc50, induced with M-CSF, RANKL, and 30 ng/ml myostatin.

The decreased expressions of TRAF6, CTR, TRAP, MMP-9, and cathepsin K indicated that overexpressed Ccdc50 inhibited osteoclast differentiation (Figure 6A). The expression levels of Ccdc50 (Figure 6B), p65, p50, and IκBa in the above-mentioned six groups indicated that myostatin suppressed the phosphorylation of p65, p50, and IκBa, which are crucial markers of the NF-κB pathway. The effects of myostatin were inhibited when Ccdc50 was overexpressed, and the silencing of Ccdc50 enhanced the effects of myostatin on the NF-κB pathway (Figure 6C). The MAPK pathway was also promoted by myostatin, as the phosphorylation of JNK, ERK, and p38 was promoted in the process. All these results showed that Ccdc50 inhibited the relative effects of myostatin (Figure 6D).

## Discussion

In this study, we demonstrated that myostatin promotes osteoclastogenesis under the regulation of Ccdc50 *in vitro*. By its connection with ActR II B, myostatin promotes activation of NF-κB and MAPK pathways. This process requires Smad2 to mediate the downstream signals, including NF-κB and MAPKs, as the inhibition of Smad2 suppressed the activation of the two osteoclastogenesis-related pathways. Our experiments indicated that myostatin regulates osteoclastogenesis via expression of Ccdc50. Overexpression of Ccdc50 inhibited osteoclastogenesis, while silencing Ccdc50 enhanced osteoclastogenesis. These results indicated that Ccdc50 is essential for myostatin’s promotion of osteoclastogenesis and for NF-κB and MAPK pathways. Myostatin can potentially serve as a target for the regulation of osteoclastogenesis-related physiological and pathological processes, since it significantly promotes osteoclastogenesis and related pathways.

The process of bone remodeling is highly regulated and maintained by the dynamic equilibrium of bone formation and bone resorption. The core of this process is composed of two parts including the resorption of inorganic minerals in bone by osteoclasts and the formation of matrix and collagenous fiber by osteoblasts (Xiao et al., 2016b). In osteoporosis, the balance of osteoclasts and osteoblasts is broken, and the rate of bone resorption is increased in comparison with bone formation (Xiao et al., 2016a). Recent studies have demonstrated that myostatin may play an essential role in bone metabolism, even though its main function is negatively regulating muscle fiber growth (Bray, 2015; Tagliaferri et al., 2015). The bone density of myostatin-knockout mice is increased significantly, and bone volume is greater than that of wild-type mice by as much as 60% (Hamrick et al., 2002; Hamrick et al., 2006; Kellum et al., 2009). Likewise, after injection with myostatin inhibitors, bone mass is increased significantly in rats (Chiu et al., 2013). This indicates that myostatin is an important regulator of bone resorption. Furthermore, the inhibition of myostatin reduces bone destruction caused by osteoarthritis, so myostatin could be a target for treating osteoarthritis (Bray, 2015; Dankbar et al., 2015; Onuora, 2015). All these studies show that myostatin may participate in the activation of osteoclasts and could be a potential target for curing orthopedic diseases.

We previously found that ActR II B exists on BMMC and RAW264.7 cell membranes at low levels. However, ActR II B increases sharply in the process of osteoclastogenesis, which means that interaction of myostatin and its receptor would be enhanced during the process, and that myostatin could play an important role in osteoclastogenesis.

It has been reported that RANKL/RANK/OPG participates in the differentiation of osteoclasts (Pepene et al., 2011; Walsh and Choi, 2014). RANKL expression is stimulated in postmenopausal osteoporosis and glucocorticoid osteoporosis patients (Liu and Zhang, 2015). The current study demonstrates that myostatin promotes osteoclastogenesis and osteoclast function. In the early stage of the RANKL pathway, RANKL combines with RANK, and then tumor necrosis factor receptor-associated factor 6 (TRAF6) is recruited and activates the MAPK and NF-κB pathways (Jimi et al., 1998; Tan et al., 2017). The main function of NF-κB is to bind RANKL and transfer signals to regulate osteoclast formation (Miyazaki et al., 2000). Our results showed that phosphorylation of p65 and p50 was promoted after treatment with myostatin, which demonstrated that myostatin can promote the activation of the NF-κB pathway within a short time. Furthermore, we demonstrated the activation of MAPKs in the presence of myostatin by detecting the phosphorylation of c-JNK, ERK, and p38. Similar to the results for NF-κB, we found higher expression when induced by RANKL and myostatin than when induced by RANKL only, which indicated that myostatin promoted the activation of MAPKs.

A previous study suggested that target genes are transcribed by the phosphorylation of Smad2 after myostatin binds to ActR II B (Walsh and Celeste, 2005). Once TGF-β receptors are activated and Smad2 is phosphorylated in turn, a complex containing Smad2, Smad4, and FAST assembles in the nucleus and activates the transcription of multiple genes (Wrana and Attisano, 2000). One result of this transcriptional activation is an increase in osteoclast production and alveolar bone loss in mice (Alotaibi et al., 2016). Our study suggests that phosphorylation of Smad2 increases with the addition of myostatin in osteoclastogenesis, and a series of osteoclast-related markers increases at the same time. However, the phosphorylation of markers of NF-κB and MAPKs clearly decreased after the addition of LY209761, which means that the activation of NF-κB and MAPKs was regulated by Smad2 protein. This process connects the downstream channel of myostatin and classical osteoclast activation pathways.

Despite these results, the upstream signals that regulate myostatin in osteoclastogenesis have remained unclear. To find candidate genes that participate in osteoclastogenesis after treatment with myostatin, a CHIP assay was performed, and 20 genes that were clearly upregulated or downregulated were selected. These differentially expressed genes play important roles in inflammation (Slominski et al., 2020), calcium channels (Fenninger et al., 2019), transcription (Nizon et al., 2019; Gura et al., 2020), cell differentiation (Gobbi et al., 2019), and cell apoptosis (Morris et al., 2020). II9, for example, is critical for mast cell-driven diseases (Abdul Qayum et al., 2019), while Htatip2 can regulate the cell cycle and promote cell apoptosis (Zhao et al., 2020). Among them, we selected Ccdc50 as a gene for study. Previous studies have demonstrated that Ccdc50, which belonged to the downregulated group, is related to the NF-κB pathway (Farfsing et al., 2009; Kameda et al., 2009) and might regulate osteoclastogenesis specifically. However, the function of Ccdc50 remains largely unknown, as it also participates in other pathological and physiological processes. Our study, for the first time, proved that the enhancement of osteoclastogenesis by myostatin was associated with low expression of Ccdc50, as overexpression of Ccdc50 inhibited osteoclastogenesis and osteoclast function, while the silencing of Ccdc50 played the opposite role. The inhibition of NF-κB and MAPK pathways was also verified in osteoclastogenesis when Ccdc50 was overexpressed and vice versa. This indicated that Ccdc50 is significant for myostatin’s induction of osteoclastogenesis by acting on the NF-κB and MAPK pathways.

In summary, we found that myostatin promoted osteoclastogenesis induced by RANKL *in vitro* by activating Smad2 and further activated the NF-κB and MAPK pathways. Furthermore, the process of osteoclastogenesis through the enhancement of myostatin was inhibited by Ccdc50. These findings imply that myostatin can serve as a potential target for overactivation-related pathological processes and provide a basis for understanding the mechanism of bone remodeling in future research.

## Data Availability Statement

The raw data supporting the conclusions of this article will be made available by the authors, without undue reservation, to any qualified researcher.

## Author Contributions

LC and JC designed the study. XZ, QC, SS, HC, ZG, WW, and XC performed the experiments. WW and QZ analyzed the data. LC interpreted the data. XZ and QC wrote the manuscript.

## Funding

This work was supported by the National Natural Science Foundation of China (81701364, 81901426), Shanghai Sailing Program (19YF1447400), Changhai Hospital Initial Foundation (2018QNA012).

## Conflict of Interest

The authors declare that the research was conducted in the absence of any commercial or financial relationships that could be constructed as a potential conflict of interest.

## References

[B1] Abdul Qayum A. Koh B. Martin R. K. Kenworthy B. T. Kharwadkar R. Fu Y. (2019). The Il9 CNS-25 regulatory element controls mast cell and basophil IL-9 production. J. Immunol. 203, 1111–1121. 31350354 10.4049/jimmunol.1900272 PMC6702076

[B2] Alotaibi M. K. Kitase Y. Shuler C. F. (2016). Smad2 overexpression induces alveolar bone loss and up regulates TNF-α, and RANKL. Arch. Oral Biol. 71, 38–45. 27421098 10.1016/j.archoralbio.2016.06.023

[B3] Asagiri M. Sato K. Usami T. Ochi S. Nishina H. Yoshida H. (2005). Autoamplification of NFATc1 expression determines its essential role in bone homeostasis. J. Exp. Med. 202, 1261–1269. 16275763 10.1084/jem.20051150 PMC2213228

[B4] Battaglino R. Kim D. Fu J. Vaage B. Fu X.-Y. Stashenko P. (2002). c-myc is required for osteoclast differentiation. J. Bone Miner. Res. 17, 763–773. 10.1359/jbmr.2002.17.5.763 12009006

[B5] Benskey M. J. Manfredsson F. P. (2016). Lentivirus production and purification. Methods Mol. Biol. 1382, 107–114. 10.1007/978-1-4939-3271-9_8 26611582

[B6] Bharti A. C. Takada Y. Aggarwal B. B. (2004). Curcumin (diferuloylmethane) inhibits receptor activator of NF-κB ligand-induced NF-κB activation in osteoclast precursors and suppresses osteoclastogenesis. J. Immunol. 172, 5940–5947. 10.4049/jimmunol.172.10.5940 15128775

[B7] Bialek P. Parkington J. Li X. Gavin D. Wallace C. Zhang J. (2014). A myostatin and activin decoy receptor enhances bone formation in mice. Bone 60, 162–171. 10.1016/j.bone.2013.12.002 24333131

[B8] Boyce B. F. Xiu Y. Li J. Xing L. Yao Z. (2015). NF-κB-Mediated regulation of osteoclastogenesis. Endocrinol. Metab. 30, 35–44. 10.3803/enm.2015.30.1.35 PMC4384681 25827455

[B9] Boyce B. Yao Z. Xing L. (2009). Osteoclasts have multiple roles in bone in addition to bone resorption. Crit. Rev. Eukaryot. Gene Expr. 19, 171–180. 10.1615/critreveukargeneexpr.v19.i3.10 19883363 PMC2856465

[B10] Bray N. (2015). Targeting myostatin for direct joint defence. Nat. Rev. Drug Discov. 14, 677 10.1038/nrd4745 26388227

[B11] Chiu C.-S. Peekhaus N. Weber H. Adamski S. Murray E. M. Zhang H. Z. (2013). Increased muscle force production and bone mineral density in ActRIIB-Fc-treated mature rodents. J. Gerontol. A Biol. Sci. Med. Sci. 68, 1181–1192. 10.1093/gerona/glt030 23525481

[B12] Courtois G. Gilmore T. D. (2006). Mutations in the NF-κB signaling pathway: implications for human disease. Oncogene 25, 6831–6843. 17072331 10.1038/sj.onc.1209939

[B13] Dankbar B. Fennen M. Brunert D. Hayer S. Frank S. Wehmeyer C. (2015). Myostatin is a direct regulator of osteoclast differentiation and its inhibition reduces inflammatory joint destruction in mice. Nat. Med. 21, 1085–1090. 10.1038/nm.3917 26236992

[B14] Farfsing A. Engel F. Seiffert M. Hartmann E. Ott G. Rosenwald A. (2009). Gene knockdown studies revealed CCDC50 as a candidate gene in mantle cell lymphoma and chronic lymphocytic leukemia. Leukemia 23, 2018–2026. 10.1038/leu.2009.144 19641524

[B15] Fenninger F. Han J. Stanwood S. R. Nohara L. L. Arora H. Choi K. B. (2019). Mutation of an L-type calcium channel gene leads to T lymphocyte dysfunction. Front. Immunol. 10, 2473 10.3389/fimmu.2019.02473 31736943 PMC6833481

[B16] Gobbi S. Hu Q. Foschi G. Catanzaro E. Belluti F. Rampa A. (2019). Benzophenones as xanthone-open model CYP11B1 inhibitors potentially useful for promoting wound healing. Bioorg. Chem. 86, 401–409. 10.1016/j.bioorg.2019.01.066 30769265

[B17] Gura M. A. Mikedis M. M. Seymour K. A. De Rooij D. G. Page D. C. Freiman R. N. (2020). Dynamic and regulated TAF gene expression during mouse embryonic germ cell development. PLoS Genet. 16, e1008515 10.1371/journal.pgen.1008515 31914128 PMC7010400

[B18] Hagemann C. Blank J. L. (2001). The ups and downs of MEK kinase interactions. Cell. Signal. 13, 863–875. 10.1016/s0898-6568(01)00220-0 11728826

[B19] Hamrick M. W. (2003). Increased bone mineral density in the femora of GDF8 knockout mice. Anat. Rec. A Discov. Mol. Cell Evol. Biol. 272, 388–391. 10.1002/ar.a.10044 12704695

[B20] Hamrick M. W. Mcpherron A. C. Lovejoy C. O. (2002). Bone mineral content and density in the humerus of adult myostatin-deficient mice. Calcif. Tissue Int. 71, 63–68. 10.1007/s00223-001-1109-8 12060865

[B21] Hamrick M. W. Samaddar T. Pennington C. Mccormick J. (2006). Increased muscle mass with myostatin deficiency improves gains in bone strength with exercise. J. Bone Miner. Res. 21, 477–483. 10.1016/s0736-0266(03)00105-0 16491296

[B22] Han H. Q. Zhou X. Mitch W. E. Goldberg A. L. (2013). Myostatin/activin pathway antagonism: molecular basis and therapeutic potential. Int. J. Biochem. Cell Biol. 45, 2333–2347. 10.1016/j.biocel.2013.05.019 23721881

[B23] Jimi E. Nakamura I. Ikebe T. Akiyama S. Takahashi N. Suda T. (1998). Activation of NF-κB is involved in the survival of osteoclasts promoted by interleukin-1. J. Biol. Chem. 273, 8799–8805. 10.1074/jbc.273.15.8799 9535858

[B24] Kameda H. Watanabe M. Bohgaki M. Tsukiyama T. Hatakeyama S. (2009). Inhibition of NF-κB signaling via tyrosine phosphorylation of Ymer. Biochem. Biophys. Res. Commun. 378, 744–749. 10.1016/j.bbrc.2008.11.102 19059208

[B25] Kellum E. Starr H. Arounleut P. Immel D. Fulzele S. Wenger K. (2009). Myostatin (GDF-8) deficiency increases fracture callus size, Sox-5 expression, and callus bone volume. Bone 44, 17–23. 10.1016/j.bone.2008.08.126 18852073 PMC2648293

[B26] Kular J. Tickner J. Chim S. M. Xu J. (2012). An overview of the regulation of bone remodelling at the cellular level. Clin. Biochem. 45, 863–873. 10.1016/j.clinbiochem.2012.03.021 22465238

[B27] Lee K. Seo I. Choi M. H. Jeong D. (2018). Roles of mitogen-activated protein kinases in osteoclast biology. Int. J. Mol. Sci. 19, 3004 10.3390/ijms19103004 PMC6213329 30275408

[B28] Lee S.-H. Rho J. Jeong D. Sul J.-Y. Kim T. Kim N. (2006). v-ATPase V0 subunit d2-deficient mice exhibit impaired osteoclast fusion and increased bone formation. Nat. Med. 12, 1403–1409. 10.1038/nm1514 17128270

[B29] Liu W. Zhang X. (2015). Receptor activator of nuclear factor-κB ligand (RANKL)/RANK/osteoprotegerin system in bone and other tissues (review). Mol. Med. Rep. 11, 3212–3218. 10.3892/mmr.2015.3152 25572286

[B30] Miyazaki T. Katagiri H. Kanegae Y. Takayanagi H. Sawada Y. Yamamoto A. (2000). Reciprocal role of ERK and nf-κb pathways in survival and activation of osteoclasts. J. Cell Biol. 148, 333–342. 10.1083/jcb.148.2.333 10648566 PMC2174281

[B31] Mizukami J. Takaesu G. Akatsuka H. Sakurai H. Ninomiya-Tsuji J. Matsumoto K. (2002). Receptor activator of NF-κB ligand (RANKL) activates TAK1 mitogen-activated protein kinase kinase kinase through a signaling complex containing RANK, TAB2, and TRAF6. Mol. Cell. Biol. 22, 992–1000. 10.1128/mcb.22.4.992-1000.2002 11809792 PMC134634

[B32] Morris A. B. Farley C. R. Pinelli D. F. Adams L. E. Cragg M. S. Boss J. M. (2020). Signaling through the inhibitory Fc receptor FcγRIIB induces CD8+ T cell apoptosis to limit T cell immunity. Immunity 52, 136–150. 10.1016/j.immuni.2019.12.006 31940267 PMC7326381

[B33] Nissinen T. A. Degerman J. Rasanen M. Poikonen A. R. Koskinen S. Mervaala E. (2016). Systemic blockade of ACVR2B ligands prevents chemotherapy-induced muscle wasting by restoring muscle protein synthesis without affecting oxidative capacity or atrogenes. Sci. Rep. 6, 32695 10.1038/srep32695 27666826 PMC5036092

[B34] Nizon M. Laugel V. Flanigan K. M. Pastore M. Waldrop M. A. Rosenfeld J. A. (2019). Variants in MED12L, encoding a subunit of the mediator kinase module, are responsible for intellectual disability associated with transcriptional defect. Genet. Med. 21, 2713–2722. 10.1038/s41436-019-0557-3 31155615 PMC7243155

[B35] Onuora S. (2015). Cartilage matrix stiffness regulates chondrocyte metabolism and OA pathogenesis. Nat. Rev. Rheumatol. 11, 504 10.1038/nrrheum.2015.113 26241187

[B36] Pepene C. E. Ilie I. R. Marian I. Duncea I. (2011). Circulating osteoprotegerin and soluble receptor activator of nuclear factor κB ligand in polycystic ovary syndrome: relationships to insulin resistance and endothelial dysfunction. Eur. J. Endocrinol. 164, 61–68. 10.1530/eje-10-0720 20974706

[B37] Sakuma T. Barry M. A. Ikeda Y. (2012). Lentiviral vectors: basic to translational. Biochem. J. 443, 603–618. 10.1042/bj20120146 22507128

[B38] Slominski R. M. Tuckey R. C. Manna P. R. Jetten A. M. Postlethwaite A. Raman C. (2020). Extra-adrenal glucocorticoid biosynthesis: implications for autoimmune and inflammatory disorders. Genes Immun. 21, 150–168. 10.1038/s41435-020-0096-6 32203088 PMC7276297

[B39] Sun Y. Liu W.-Z. Liu T. Feng X. Yang N. Zhou H.-F. (2015). Signaling pathway of MAPK/ERK in cell proliferation, differentiation, migration, senescence and apoptosis. J. Recept. Signal Transduct. Res. 35, 600–604. 10.3109/10799893.2015.1030412 26096166

[B40] Tagliaferri C. Wittrant Y. Davicco M.-J. Walrand S. Coxam V. (2015). Muscle and bone, two interconnected tissues. Ageing Res. Rev. 21, 55–70. 10.1016/j.arr.2015.03.002 25804855

[B41] Takayanagi H. (2007). Osteoclast differentiation and activation. Clin. Calcium 17, 484–492 [in Japanese, with English summary]. doi:CliCa0704484492. 17404476

[B42] Takayanagi H. Kim S. Koga T. Nishina H. Isshiki M. Yoshida H. (2002). Induction and activation of the transcription factor NFATc1 (NFAT2) integrate RANKL signaling in terminal differentiation of osteoclasts. Dev. Cell 3, 889–901. 10.1016/s1534-5807(02)00369-6 12479813

[B43] Tan E. M. Li L. Indran I. R. Chew N. Yong E.-L. (2017). TRAF6 mediates suppression of osteoclastogenesis and prevention of ovariectomy-induced bone loss by a novel prenylflavonoid. J. Bone Miner. Res. 32, 846–860. 10.1002/jbmr.3031 27813153

[B44] Tanos T. Marinissen M. J. Leskow F. C. Hochbaum D. Martinetto H. Gutkind J. S. (2005). Phosphorylation of c-Fos by members of the p38 MAPK family. J. Biol. Chem. 280, 18842–18852. 10.1074/jbc.m500620200 15708845

[B45] Walsh F. S. Celeste A. J. (2005). Myostatin: a modulator of skeletal-muscle stem cells. Biochem. Soc. Trans. 33, 1513–1517. 10.1042/bst0331513 16246158

[B46] Walsh M. C. Choi Y. (2014). Biology of the RANKL-RANK-OPG system in immunity, bone, and beyond. Front. Immunol. 5, 511 10.3389/fimmu.2014.00511 25368616 PMC4202272

[B47] Wen J. Liu X. Qi Y. Niu F. Niu Z. Geng W. (2019). BMP3 suppresses colon tumorigenesis via ActRIIB/SMAD2-dependent and TAK1/JNK signaling pathways. J. Exp. Clin. Canc. Res. 38, 428 10.1186/s13046-019-1435-1 PMC6819484 31665064

[B48] Wrana J. L. Attisano L. (2000). The Smad pathway, Cytokine Growth Factor Rev. 11, 5–13. 10.1016/s1359-6101(99)00024-6 10708948

[B49] Xiao W. Li S. Pacios S. Wang Y. Graves D. T. (2016a). Bone remodeling under pathological conditions. Front. Oral Biol. 18, 17–27. 10.1159/000351896 26599114 PMC10757467

[B50] Xiao W. Wang Y. Pacios S. Li S. Graves D. T. (2016b). Cellular and molecular aspects of bone remodeling. Front. Oral Biol. 18, 9–16. 10.1159/000351895 26599113 PMC10754210

[B51] Zhao B. Chen Y. Hu S. Yang N. Liu M. Li J. (2020). Characterization of HTATIP2 and its role during hair follicle cycles in Angora rabbit. Genome 63, 179–187. 10.1139/gen-2019-0132 31917611

