# Supplementary material for: Myostatin Promotes Osteoclastogenesis by Regulating Ccdc50 Gene Expression and RANKL-Induced NF-κB and MAPK Pathways
Source: Front Pharmacol. 2020 Nov 26;11:565163. doi: 10.3389/fphar.2020.565163 (PMC7849192; doi:10.3389/fphar.2020.565163)
Supplement: Supplementary file 1 [file presentation1.pptx]

## Slide 1
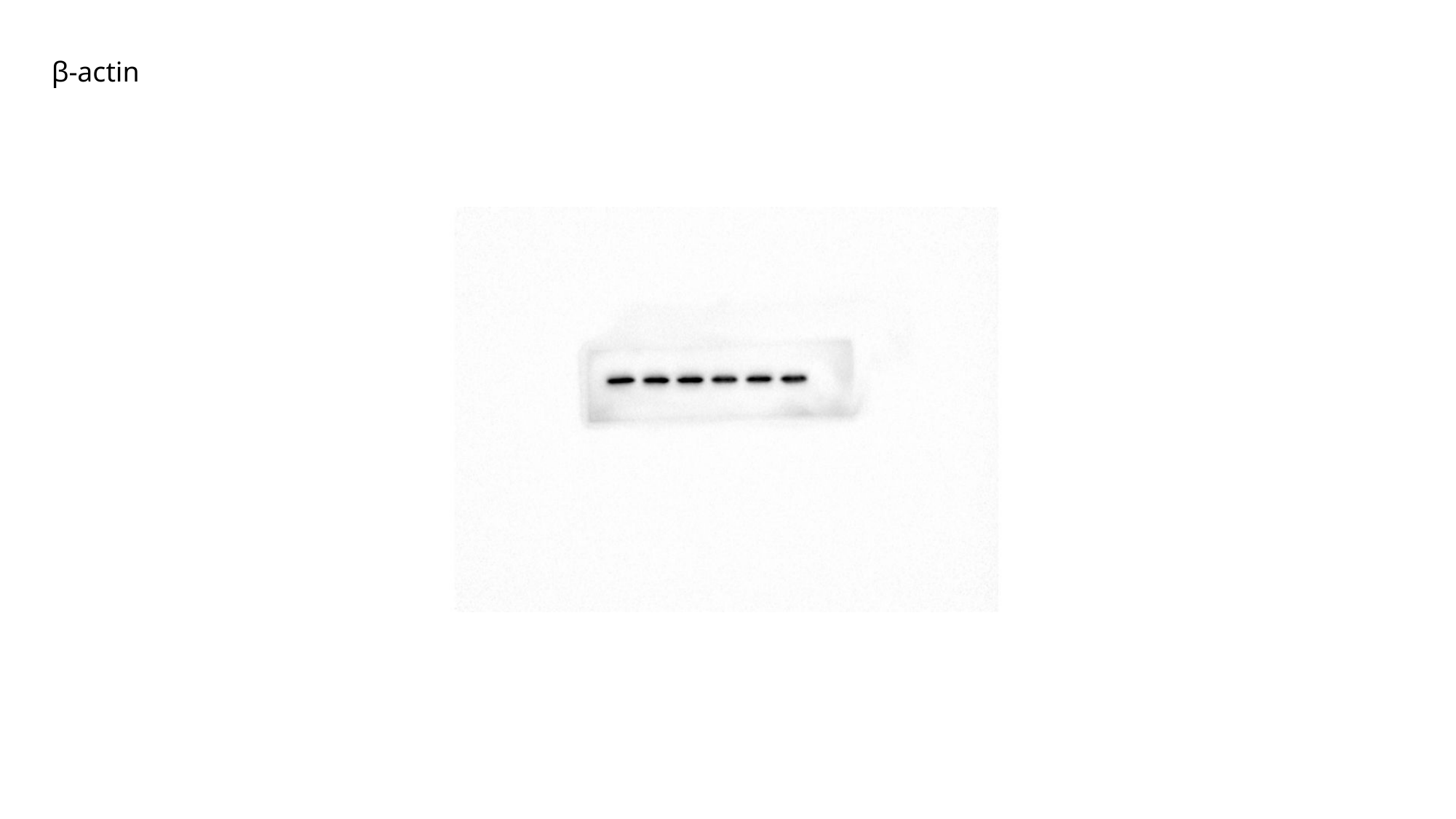

β-actin

## Slide 2
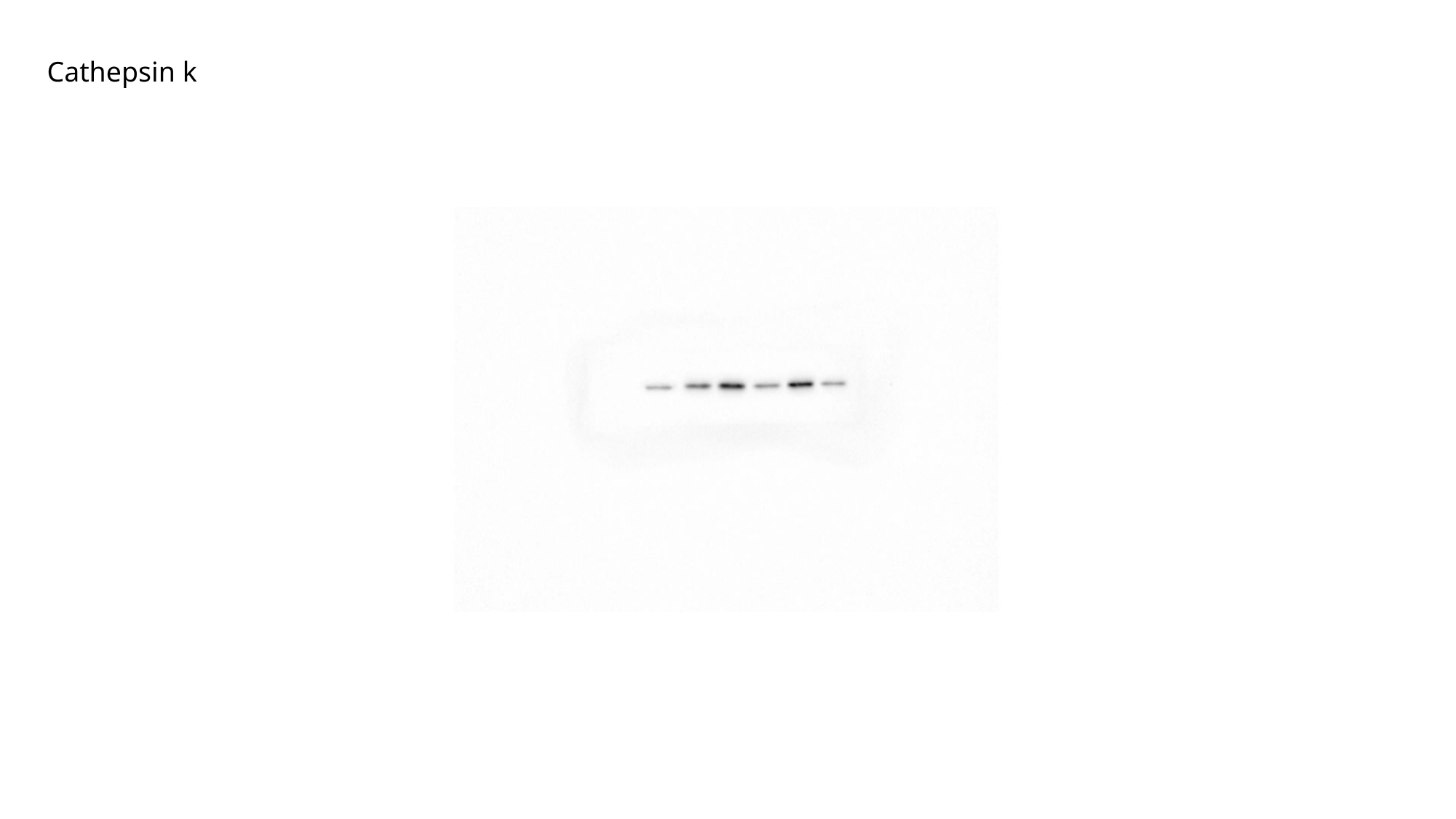

Cathepsin k

## Slide 3
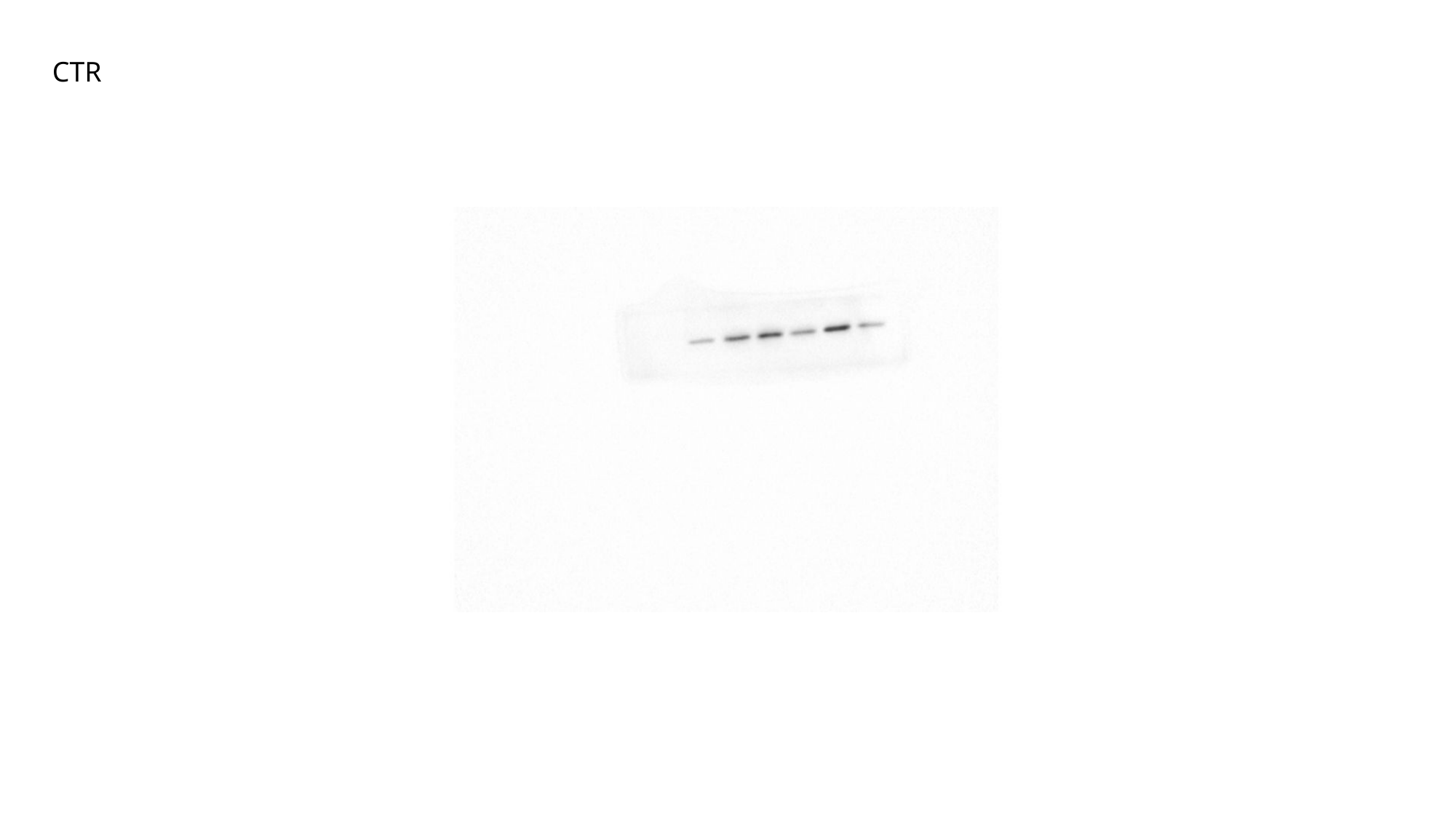

CTR

## Slide 4
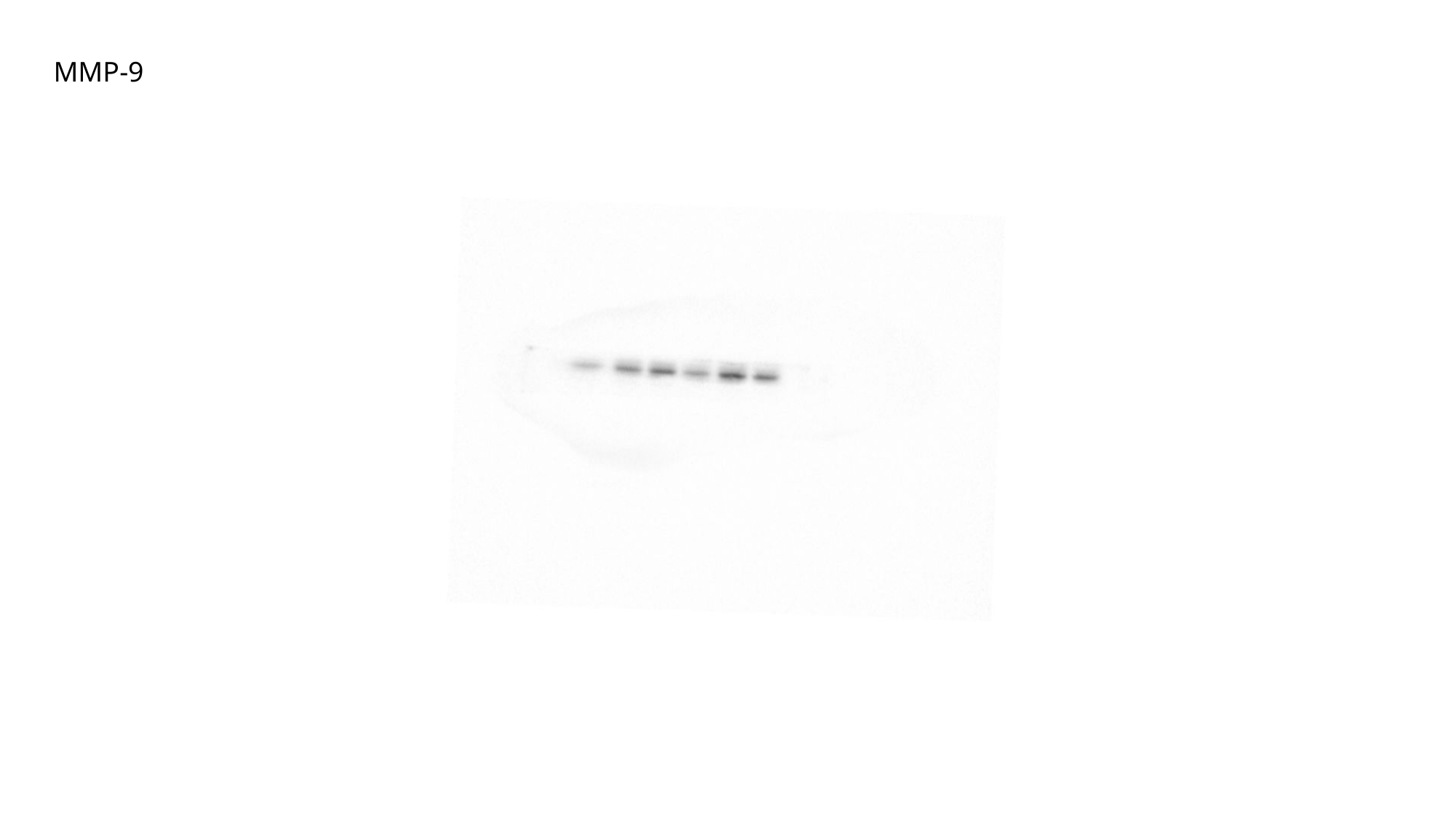

MMP-9

## Slide 5
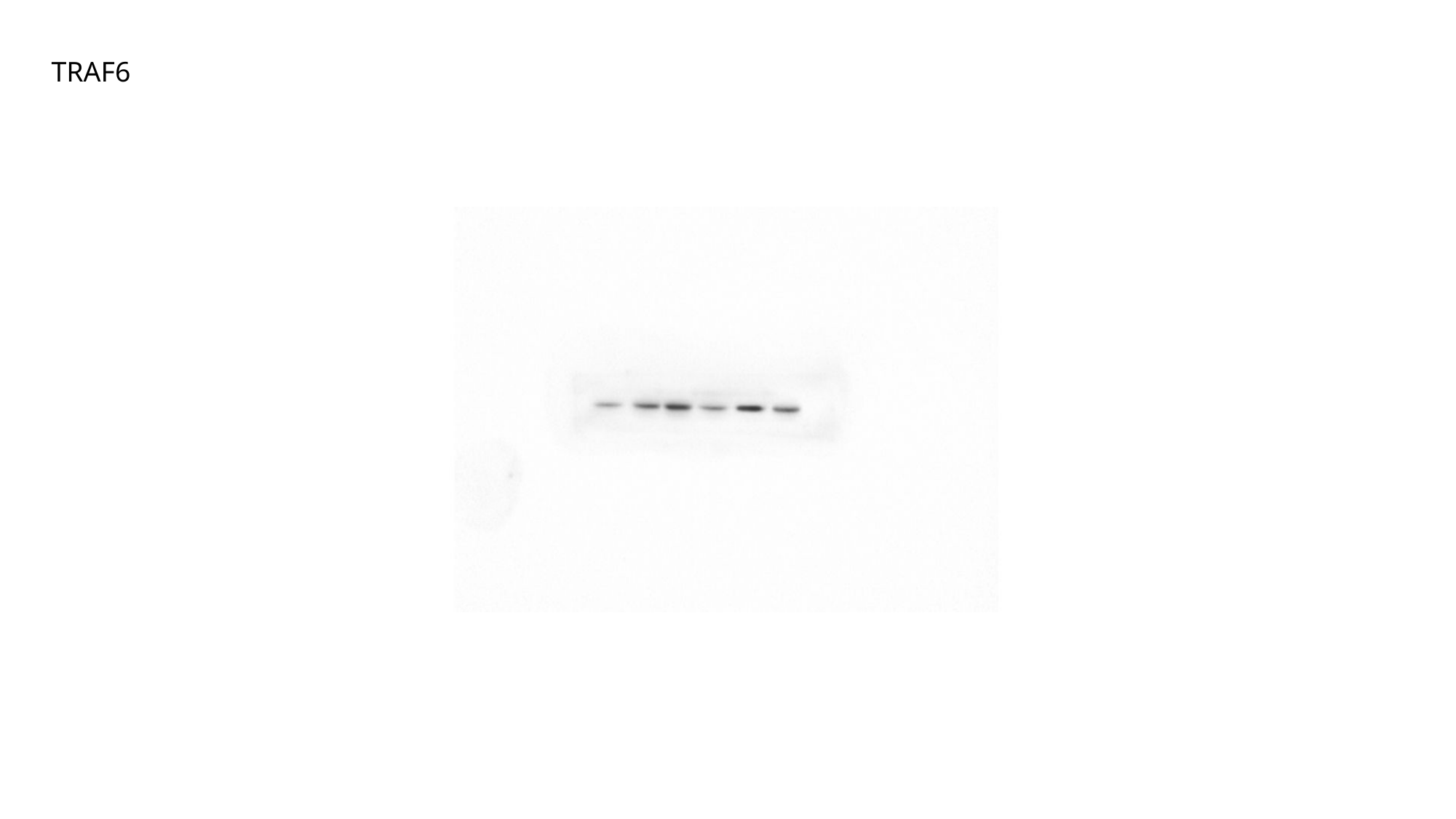

TRAF6

## Slide 6
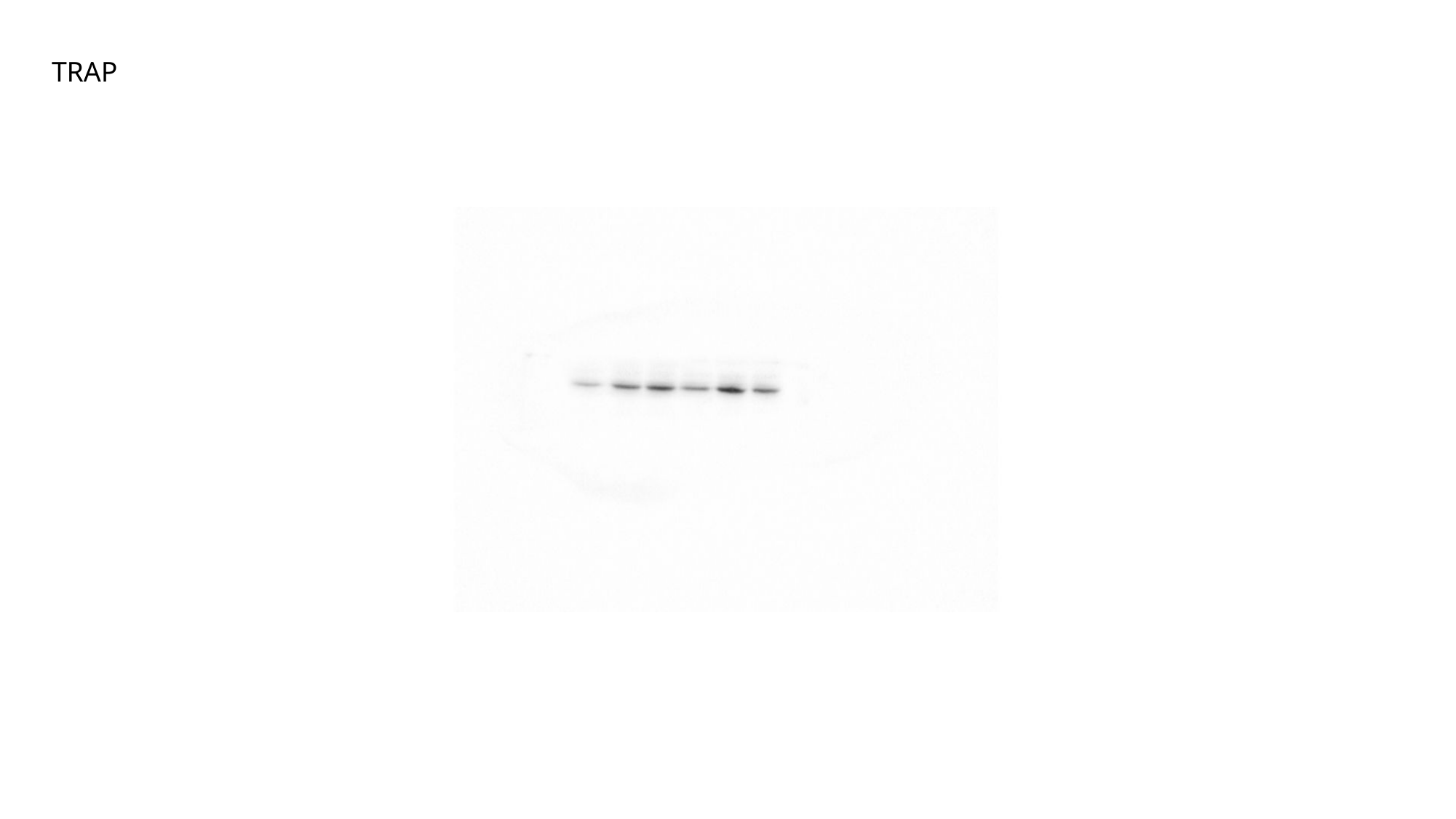

TRAP

## Slide 7
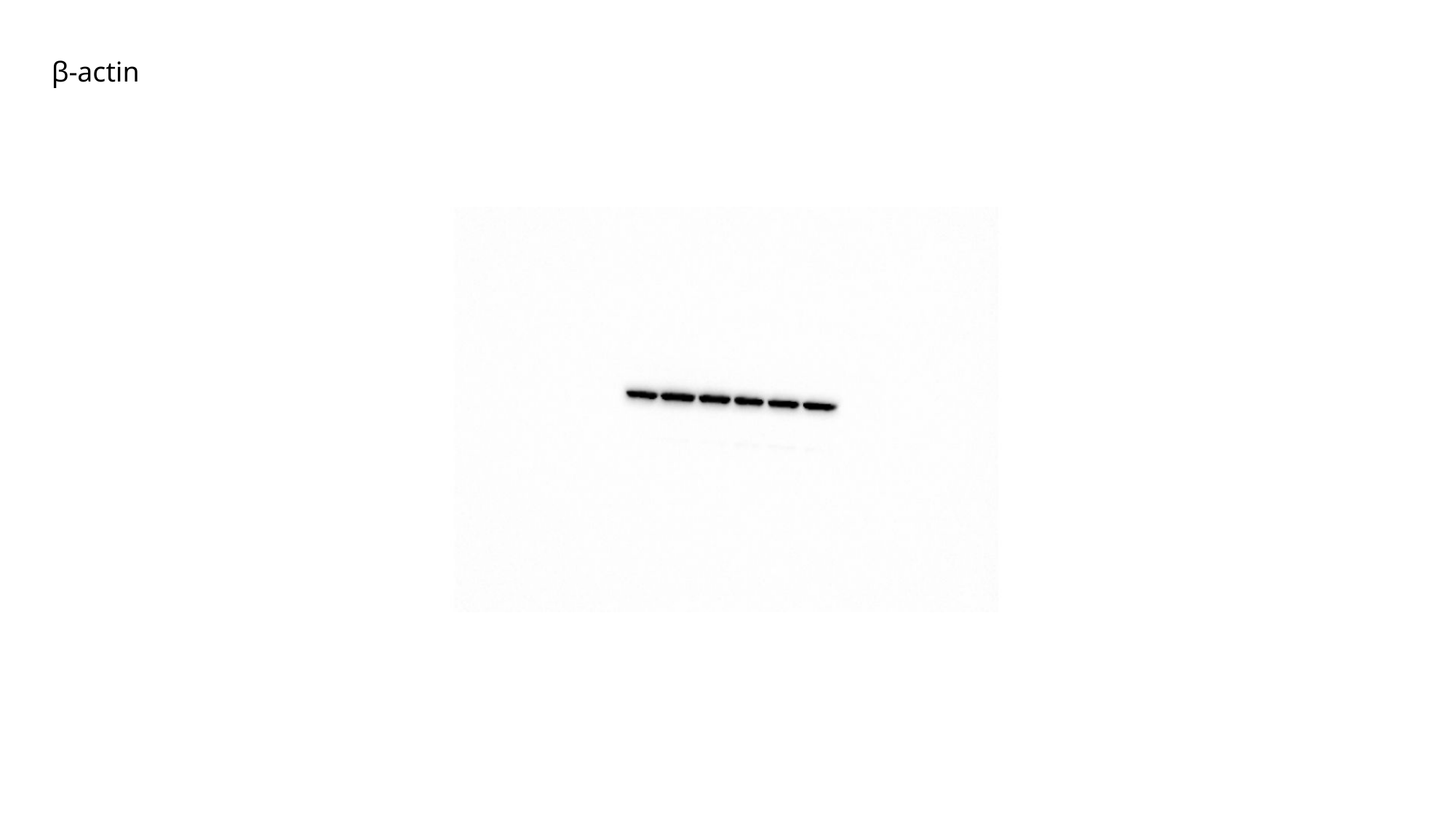

β-actin

## Slide 8
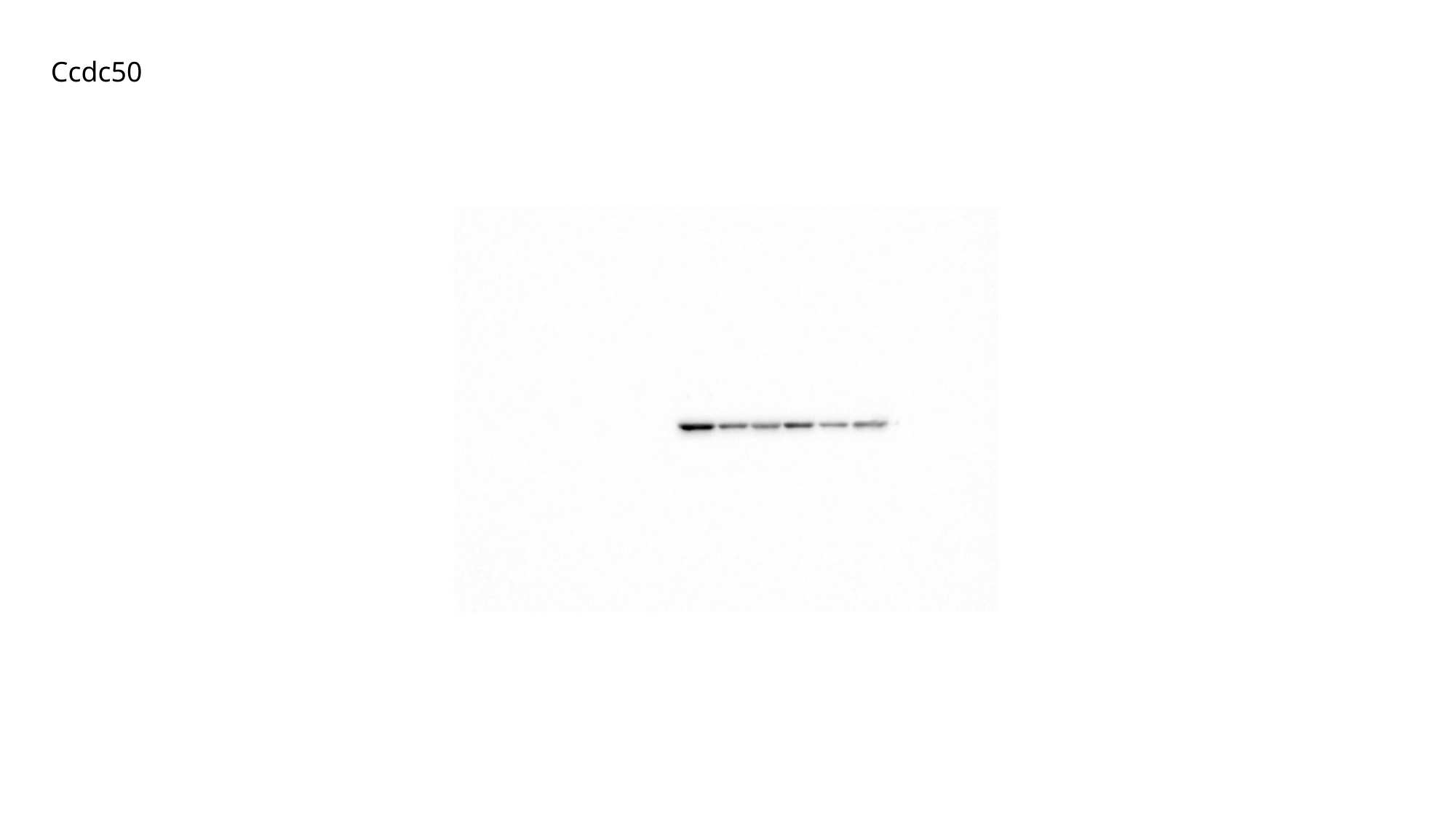

Ccdc50

## Slide 9
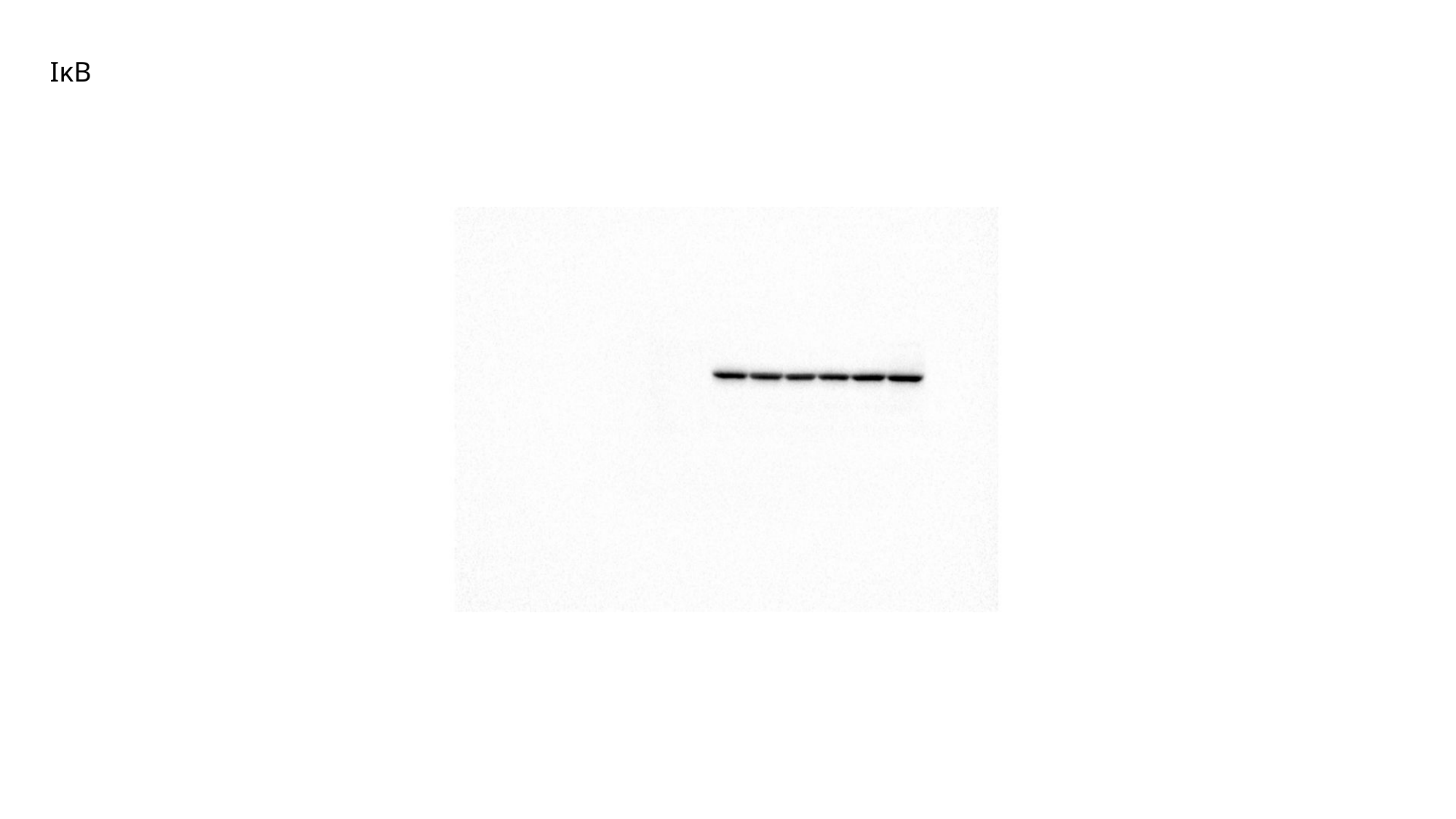

IκB

## Slide 10
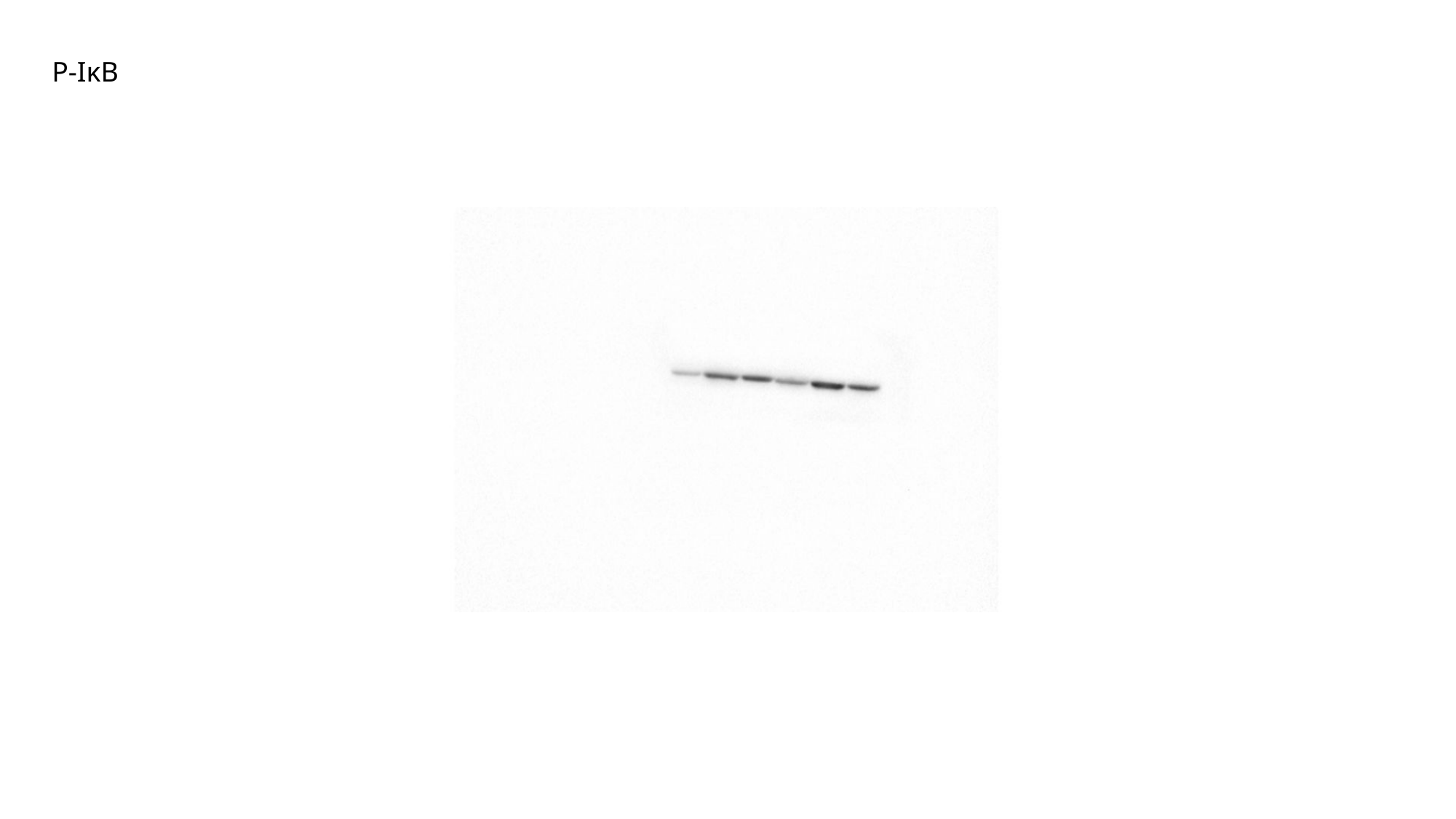

P-IκB

## Slide 11
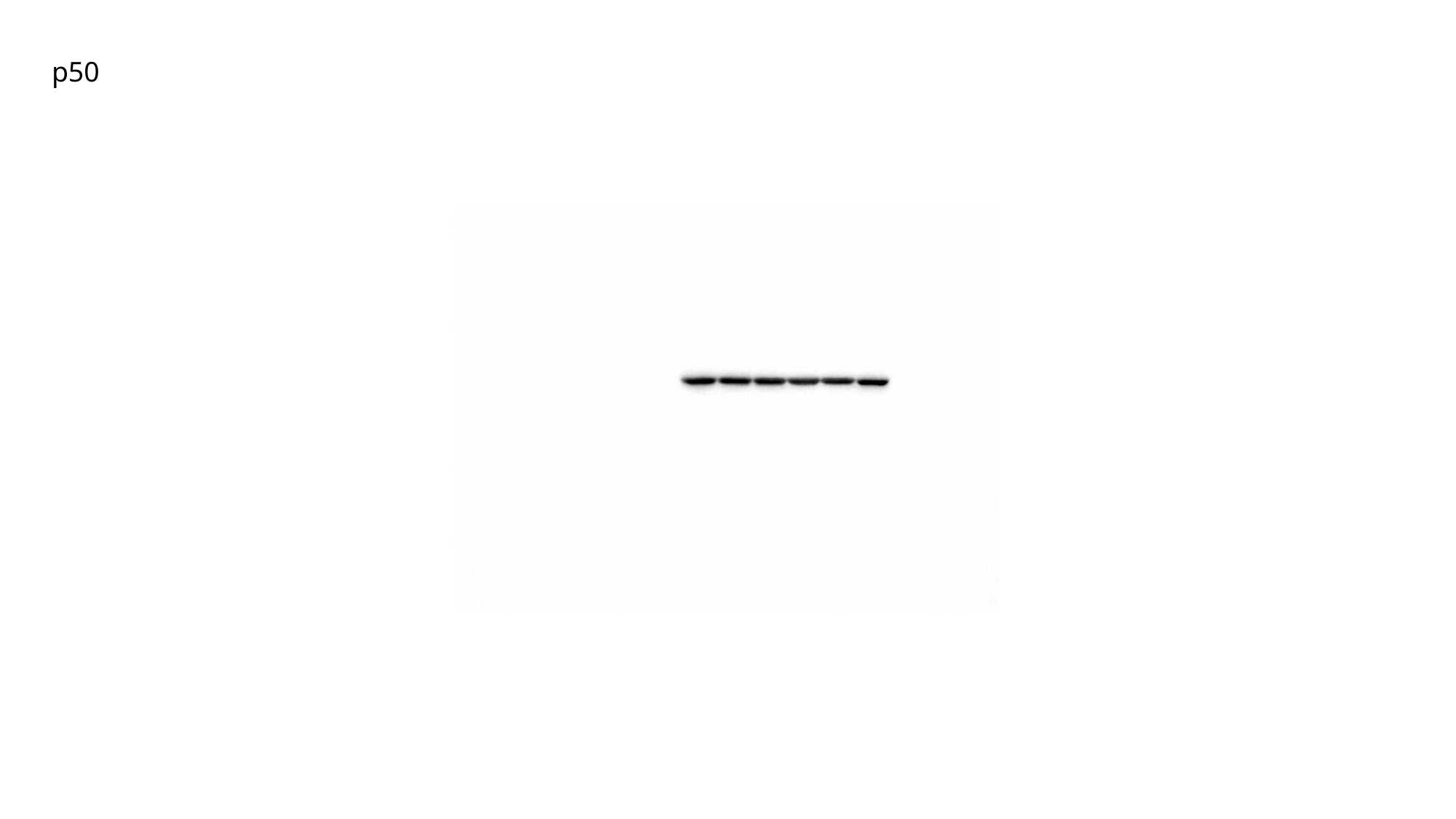

p50

## Slide 12
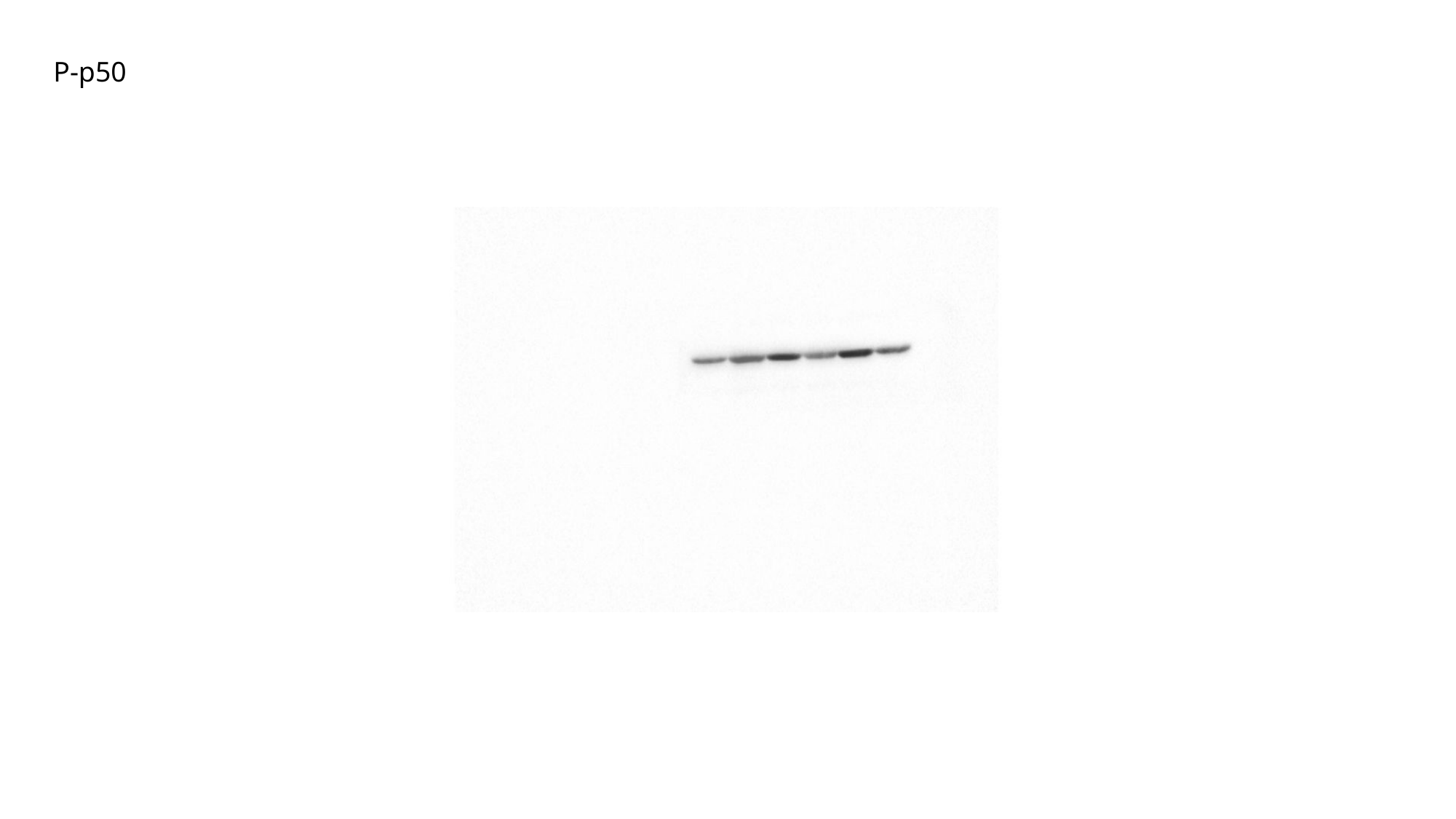

P-p50

## Slide 13
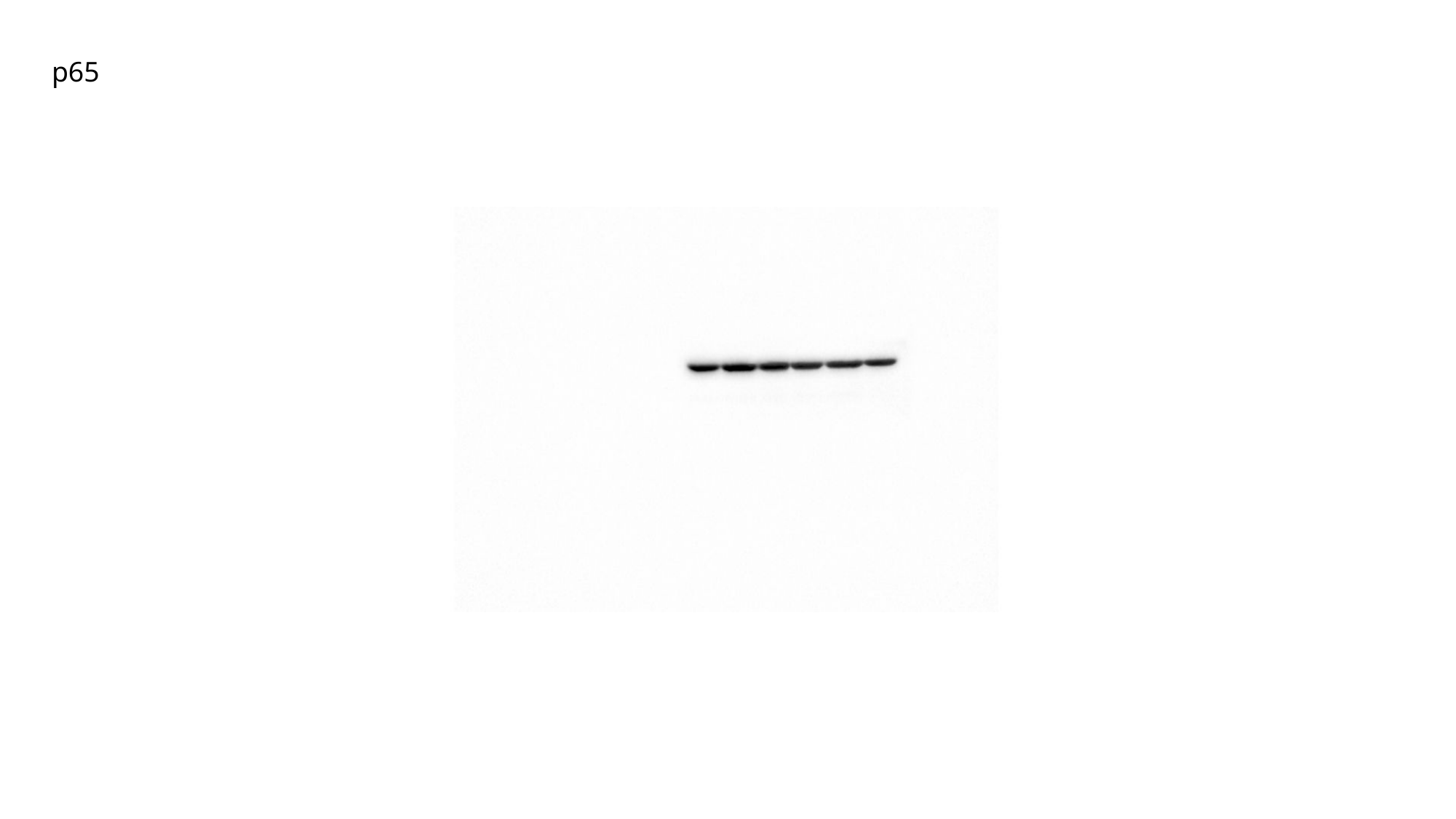

p65

## Slide 14
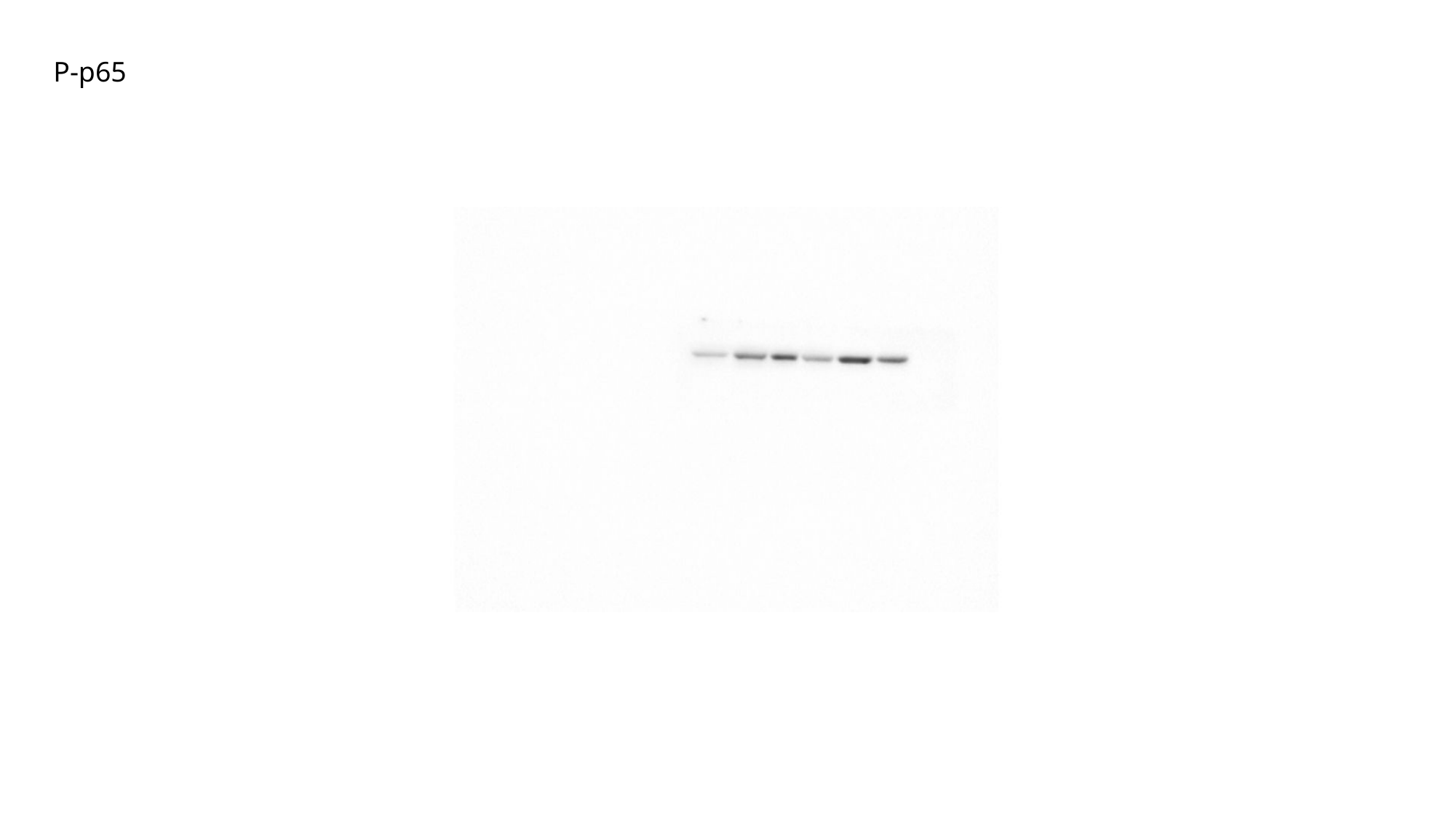

P-p65

## Slide 15
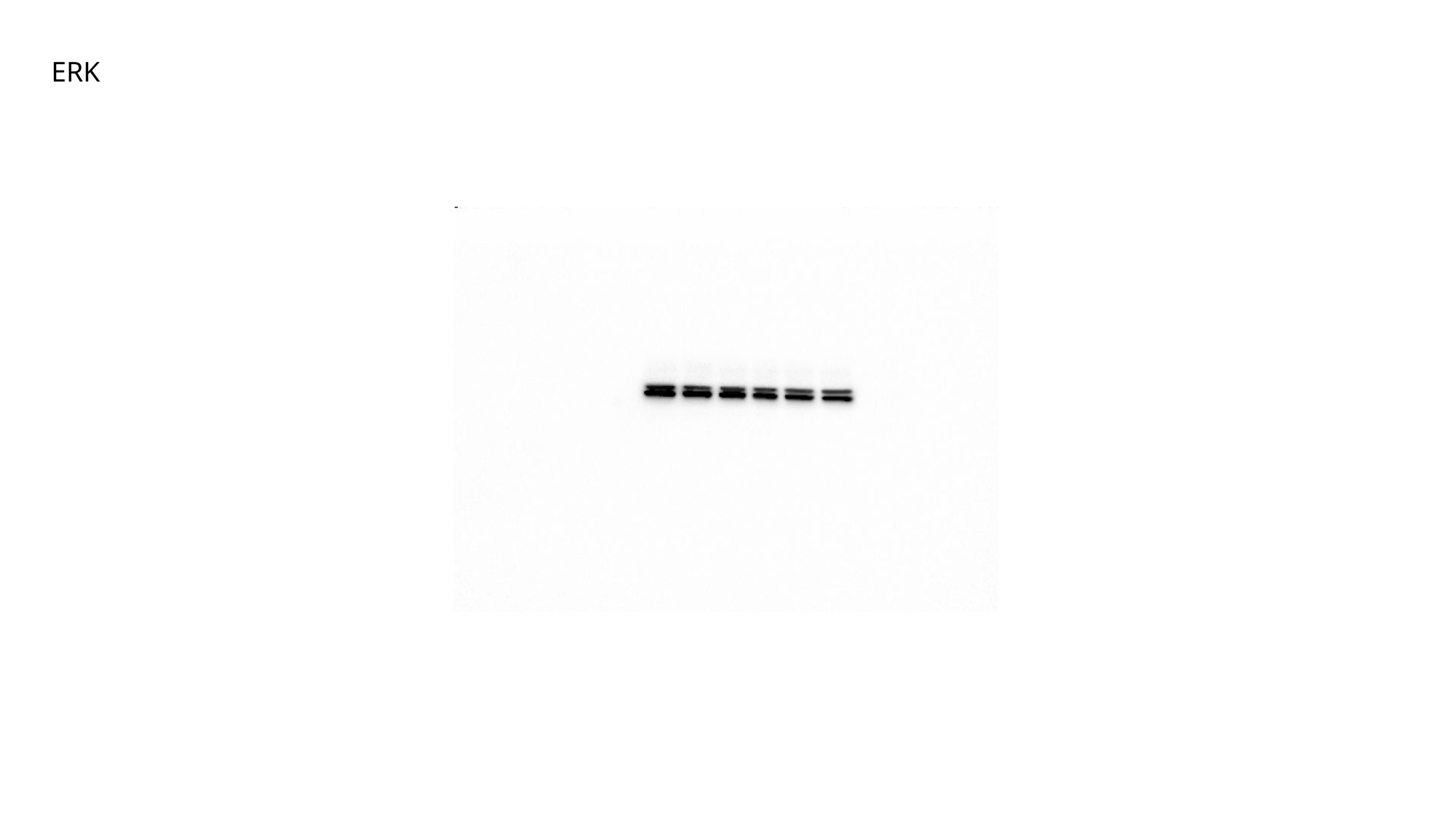

ERK

## Slide 16
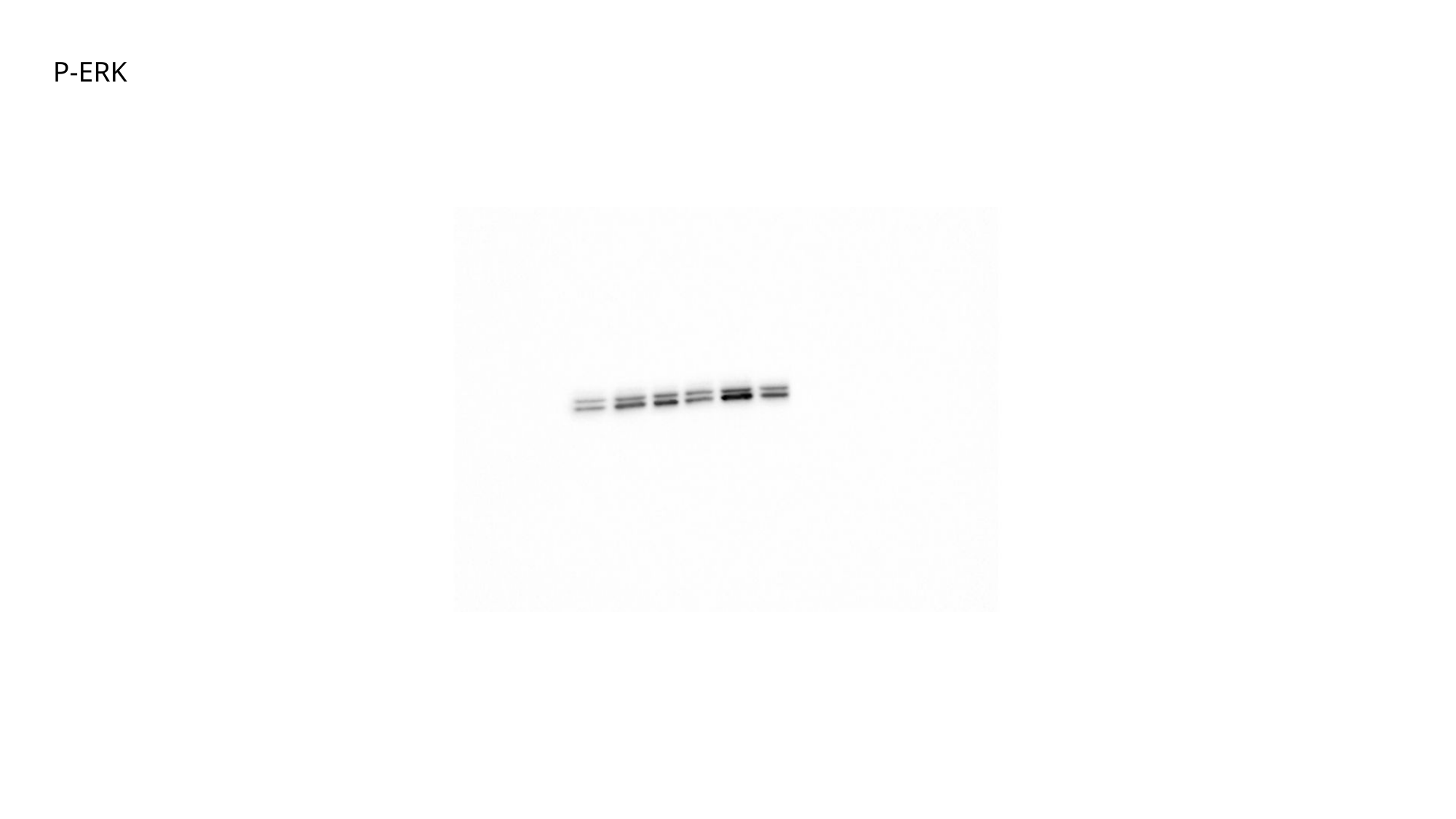

P-ERK

## Slide 17
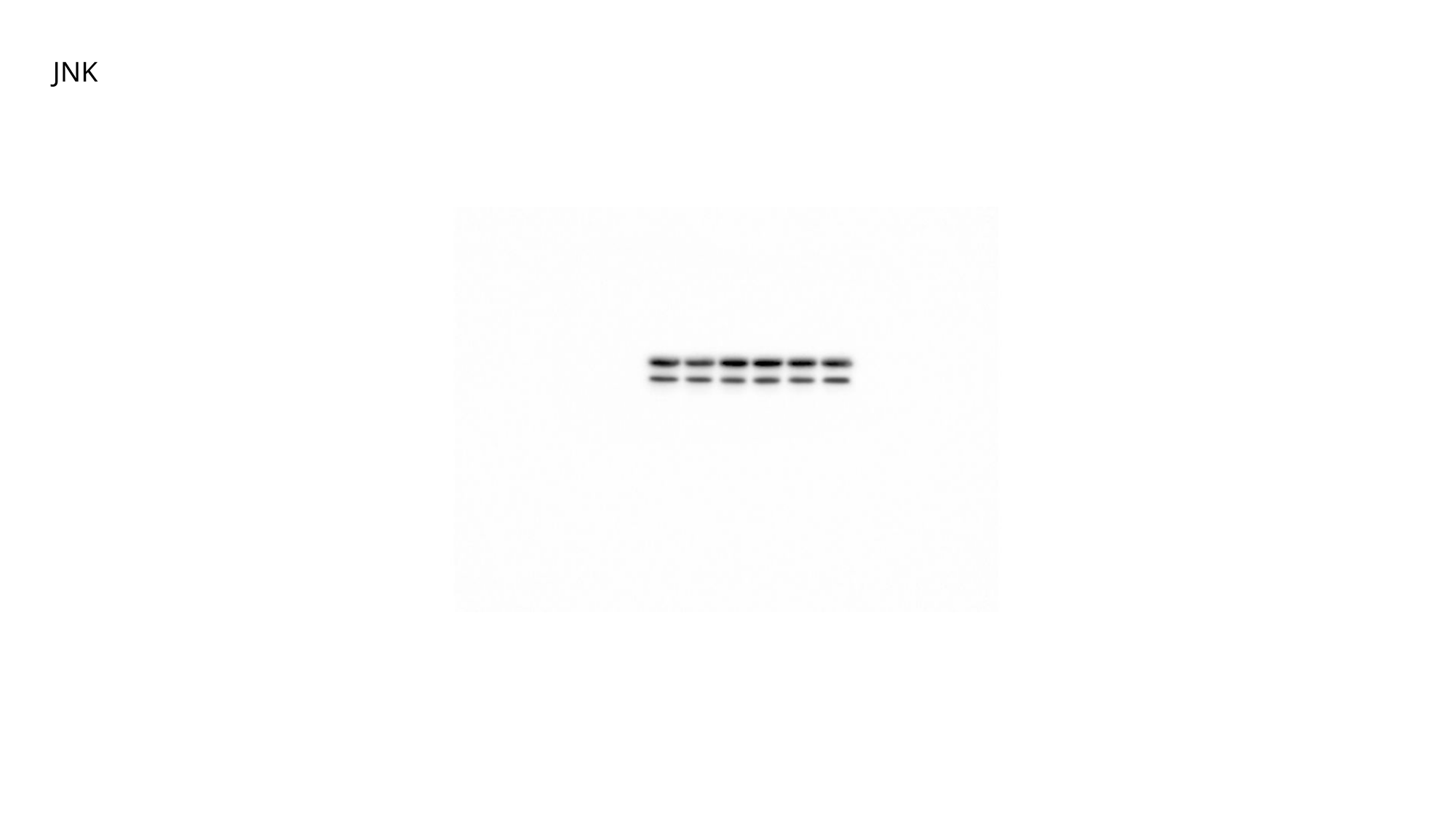

JNK

## Slide 18
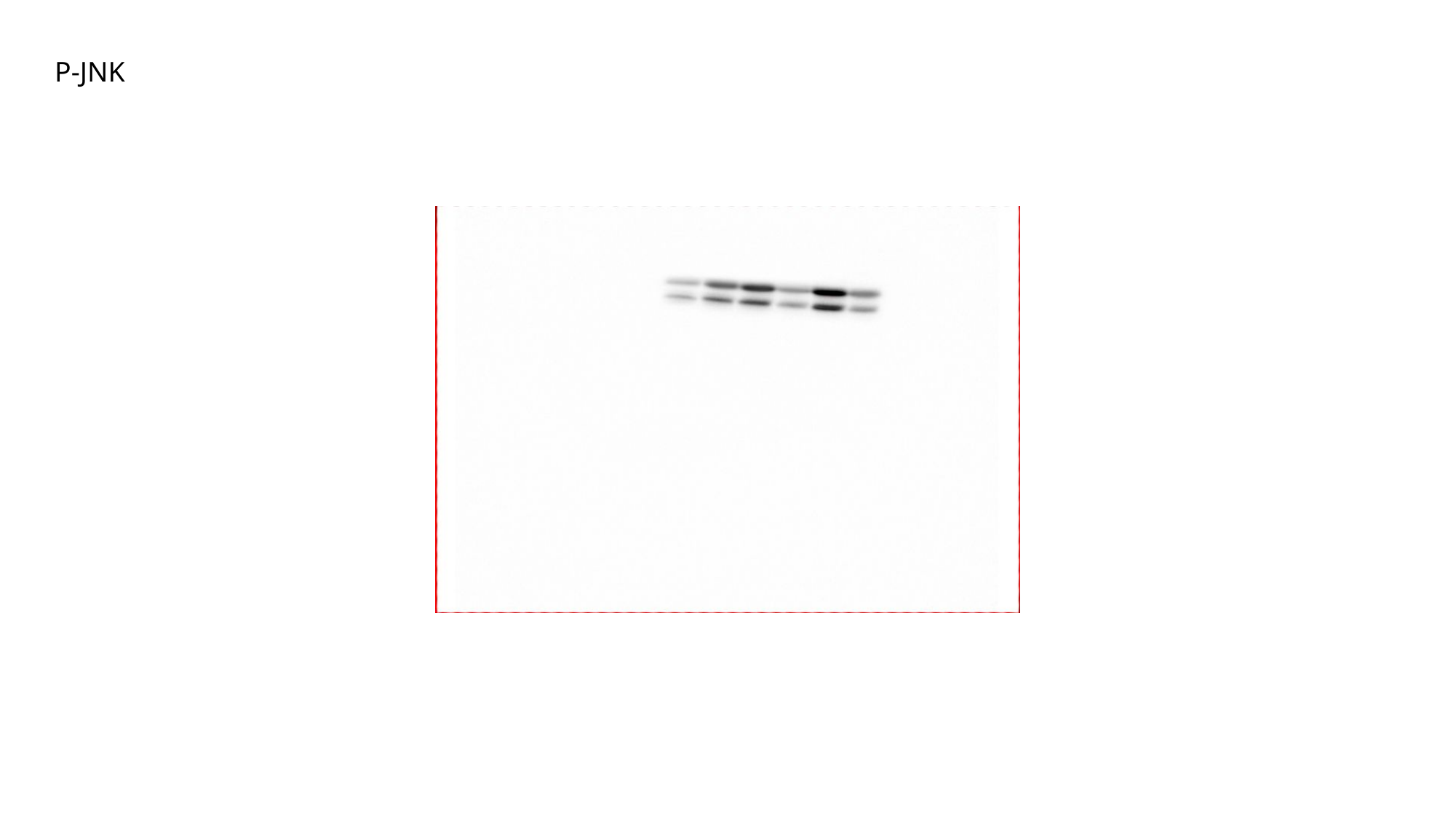

P-JNK

## Slide 19
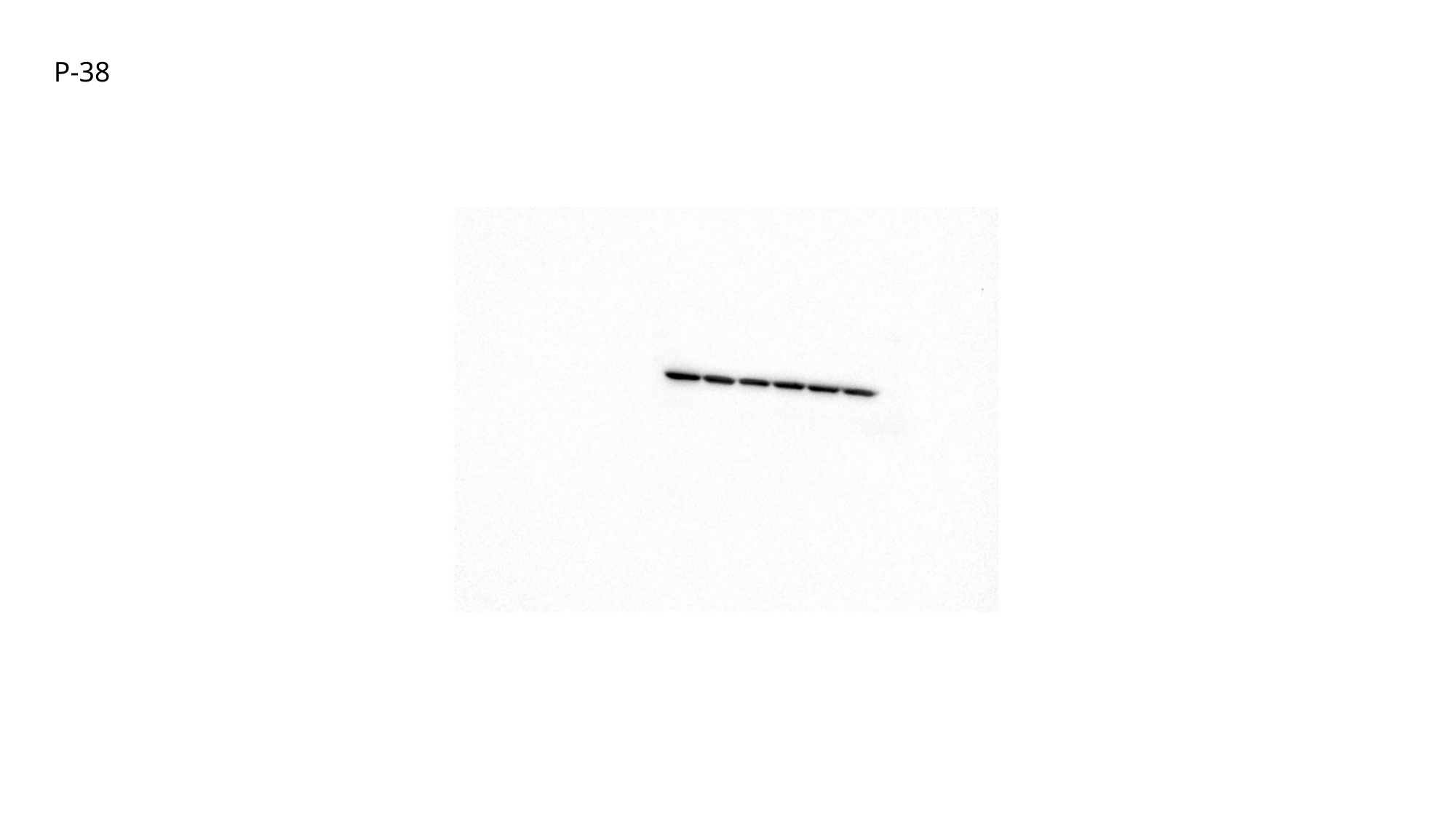

P-38

## Slide 20
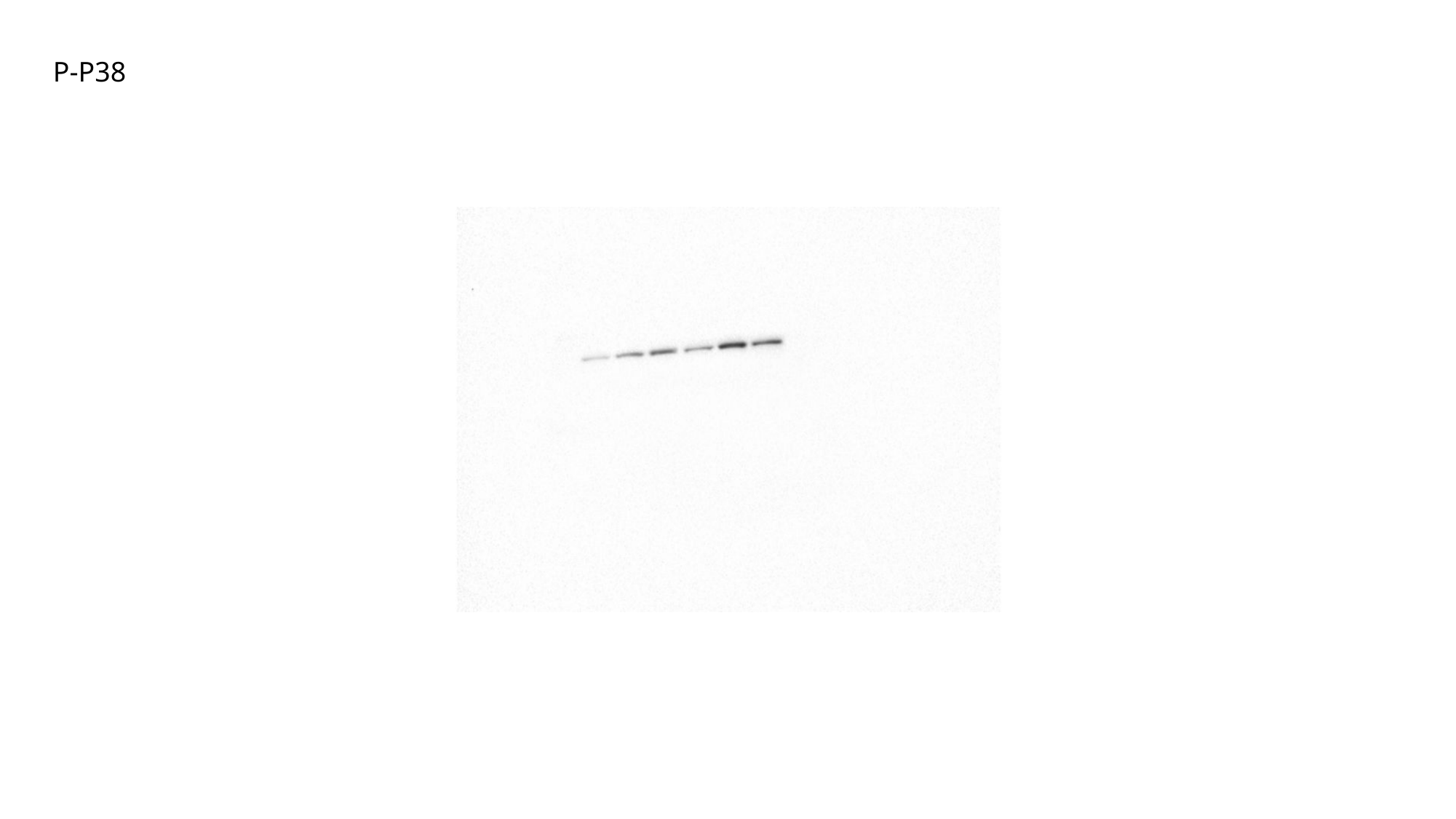

P-P38
