# Supplementary figures and images for: Myostatin Promotes Osteoclastogenesis by Regulating Ccdc50 Gene Expression and RANKL-Induced NF-κB and MAPK Pathways
Source: Front Pharmacol. 2020 Nov 26;11:565163. doi: 10.3389/fphar.2020.565163 (PMC7849192; doi:10.3389/fphar.2020.565163)

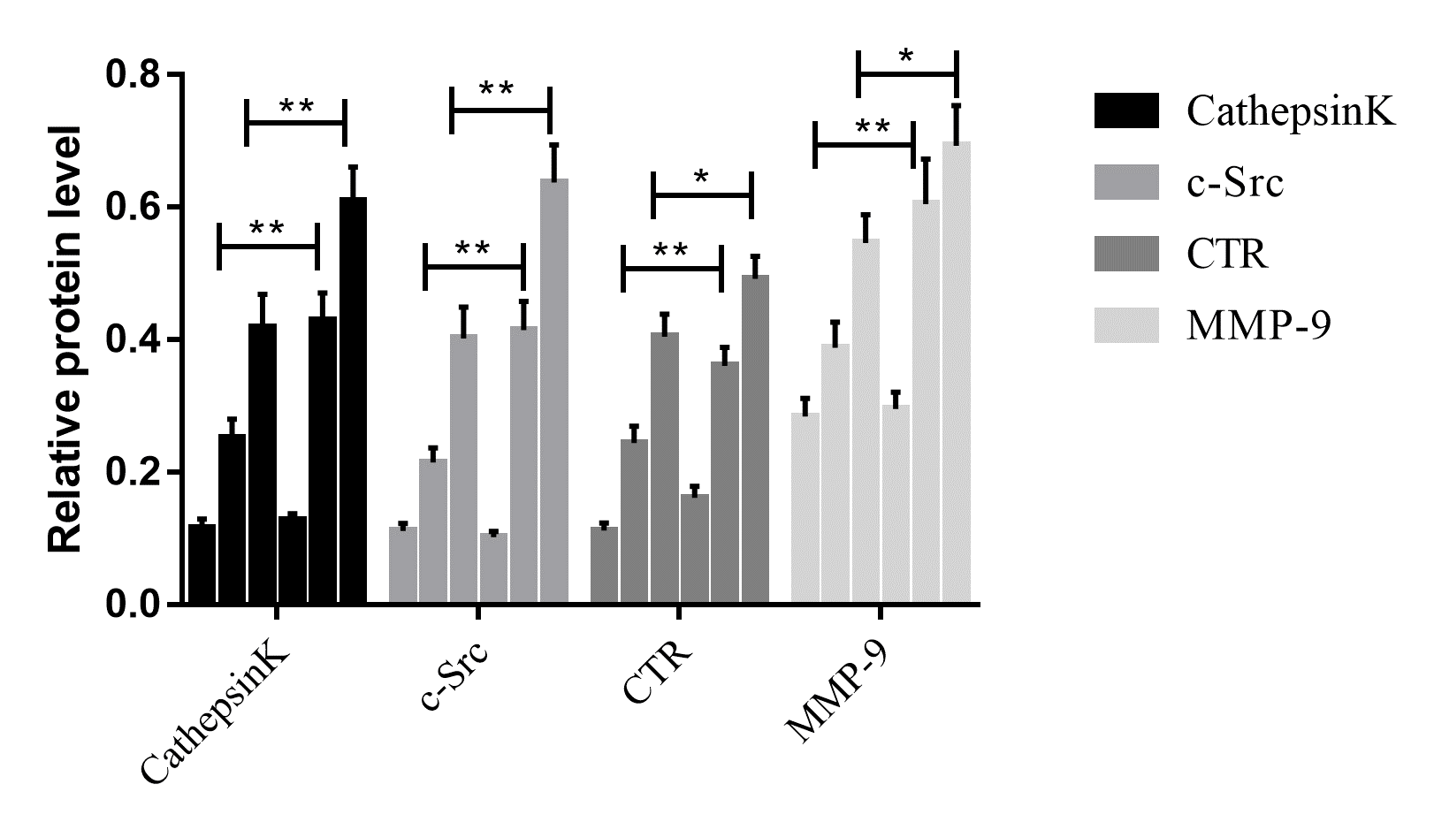

Supplement: Supplementary file 2 [file image1.tif]

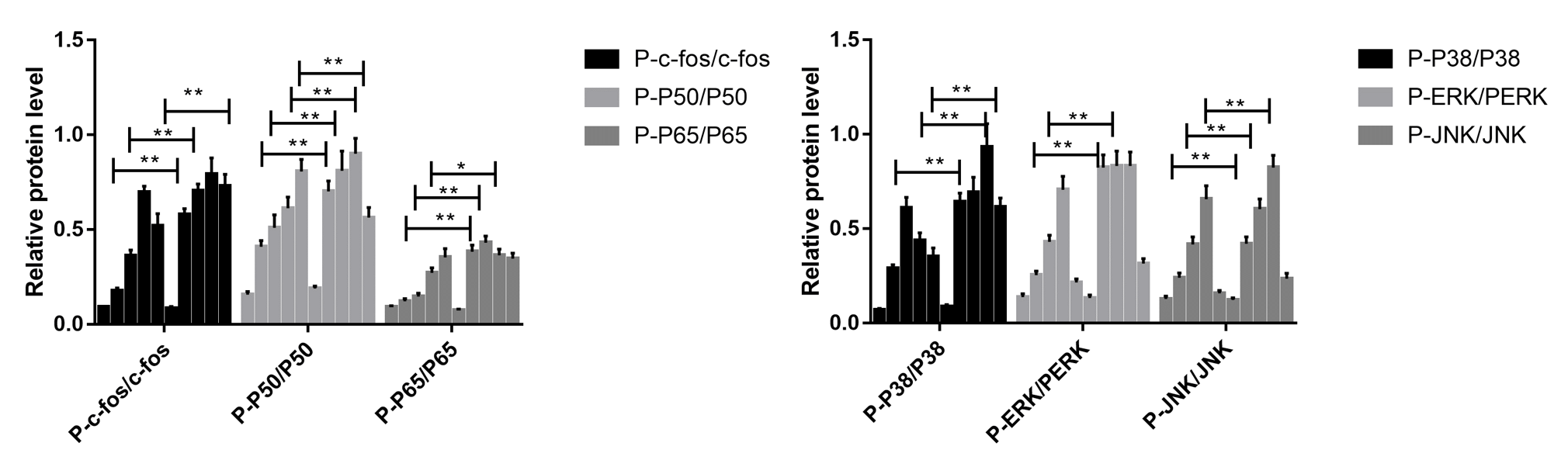

Supplement: Supplementary file 3 [file image2.tif]

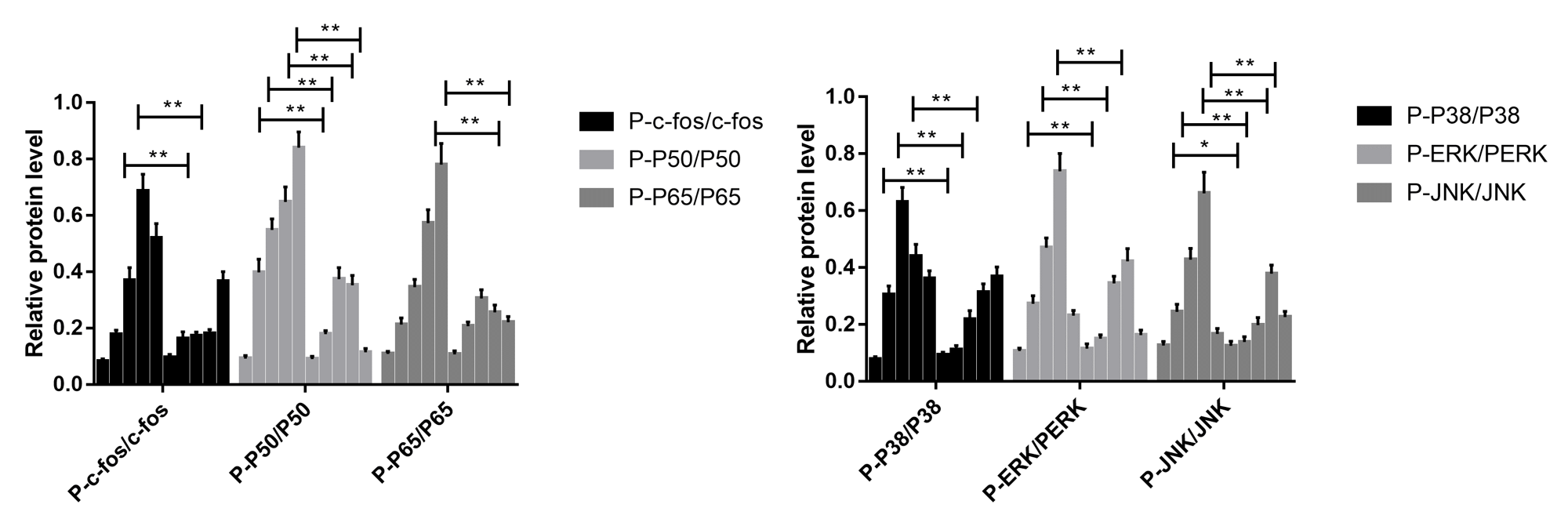

Supplement: Supplementary file 4 [file image3.tif]
